# Tipifarnib prevents development of hypoxia-induced pulmonary hypertension

**DOI:** 10.1093/cvr/cvw258

**Published:** 2017-01-05

**Authors:** Lucie Duluc, Blerina Ahmetaj-Shala, Jane Mitchell, Vahitha B. Abdul-Salam, Abdul S. Mahomed, Lulwah Aldabbous, Eduardo Oliver, Lucio Iannone, Olivier D. Dubois, Elisabeth M. Storck, Edward W. Tate, Lan Zhao, Martin R. Wilkins, Beata Wojciak-Stothard

**Affiliations:** 1 Department of Medicine, Hammersmith Campus, Imperial College London, Du Cane Road, W120NN London, UK; 2 National Heart and Lung Institute, Royal Brompton Campus, Imperial College London, Dovehouse Street, London SW3 6LY, UK; 3 Department of Chemistry, South Kensington Campus, Imperial College London, Exhibition Road, London SW7 2AZ, UK

**Keywords:** Pulmonary hypertension, Rho, Farnesylation, Endothelium

## Abstract

**Aims:**

RhoB plays a key role in the pathogenesis of hypoxia-induced pulmonary hypertension. Farnesylated RhoB promotes growth responses in cancer cells and we investigated whether inhibition of protein farnesylation will have a protective effect.

**Methods and results:**

The analysis of lung tissues from rodent models and pulmonary hypertensive patients showed increased levels of protein farnesylation. Oral farnesyltransferase inhibitor tipifarnib prevented development of hypoxia-induced pulmonary hypertension in mice. Tipifarnib reduced hypoxia-induced vascular cell proliferation, increased endothelium-dependent vasodilatation and reduced vasoconstriction of intrapulmonary arteries without affecting cell viability. Protective effects of tipifarnib were associated with inhibition of Ras and RhoB, actin depolymerization and increased eNOS expression *in vitro* and *in vivo*. Farnesylated-only RhoB (F-RhoB) increased proliferative responses in cultured pulmonary vascular cells, mimicking the effects of hypoxia, while both geranylgeranylated-only RhoB (GG-RhoB), and tipifarnib had an inhibitory effect. Label-free proteomics linked F-RhoB with cell survival, activation of cell cycle and mitochondrial biogenesis. Hypoxia increased and tipifarnib reduced the levels of F-RhoB-regulated proteins in the lung, reinforcing the importance of RhoB as a signalling mediator. Unlike simvastatin, tipifarnib did not increase the expression levels of Rho proteins.

**Conclusions:**

Our study demonstrates the importance of protein farnesylation in pulmonary vascular remodelling and provides a rationale for selective targeting of this pathway in pulmonary hypertension.

## 1. Introduction

Pulmonary hypertension (PH) is characterized by increased pulmonary vascular resistance due to pulmonary vasoconstriction and vascular remodelling and hypoxia is one of the major underlying causes of the disease.^1^

Ras family of proteins, including Rho GTPases, are key regulators of actin dynamics and cell proliferation.^2^ Activation of RhoA contributes to sustained hypoxic pulmonary vasoconstriction and pulmonary vascular remodelling in hypoxia-induced PH and in other forms of PH.^3^ We have recently shown that RhoB, a protein homologous to RhoA is required for hypoxia-induced HIF-1α stabilization, vascular cell proliferation, and migration *in vitro.*^4^ Coordinated activation of RhoA and RhoB maximizes contractile responses of VSMCs to hypoxia: RhoB induces actin polymerization via its interaction with mammalian homolog of Drosophila diaphanous (mDia), while RhoA and its effector, Rho kinase, increase phosphorylation of myosin light chain on Ser19, thereby facilitating the formation of contractile actomyosin (stress) fibres.^4^ RhoB gene deletion attenuates PH in mice, indicating a unique role of this protein in the disease pathology.^4^

RhoB is distinguished from other Rho proteins by being either farnesylated (F-RhoB) or geranylgeranylated (GG-RhoB) while other Rho proteins are only geranylgeranylated.^5^ Lipid modification (isoprenylation) of Ras proteins is important for their biological activity. Attachment of the 20-carbon chain (geranylgeranylation) or 15-carbon chain (farnesylation) at the C-terminal CAAX motif enhances their binding to the cell membrane, important step for the interaction with signaling effectors. Isoprenylation is carried out by enzymes geranylgeranyl-transferases (GGTase-1 and GGTase-2) or farnesyl-transferase (FTase). Biological activity of RhoB is thought to be determined by its prenylation status.^6^ In cancer cells, geranylgeranylation of RhoB induces apoptosis and is required for RhoB degradation,^7^ while farnesylation of RhoB is associated with pro-proliferative effects.^6^

Protein prenylation can be inhibited by statins, which limit biosynthesis of the isoprenoid precursor, mevalonate. In spite of the successful outcomes in preclinical studies, statins are not effective in the treatment of human PH.^8^ This disappointing clinical outcome may result from the fact that statins do not completely inhibit protein farnesylation at safe therapeutic doses,^9^ suggesting that proteins that require farnesylation for their activity, like RhoB or Ras, may remain functional.

We hypothesized that selective inhibition of protein farnesylation will have a protective effect in pulmonary hypertension. FTase inhibitors (FTIs) have undergone extensive clinical testing in various hematologic malignancies and progeria.^10^^,^^11^ Recent Phase II clinical trials demonstrated high effectiveness of lonafarnib in reducing viral infection and treatment of chronic hepatitis delta.^12^ Orally bioavailable, non-peptidomimetic FTI tipifarnib has shown promising effects in clinical trials for the treatment of leukemia^13^ and cancer.^14^ The suitability of FTIs in treatment of PH has not been investigated, though an isolated case study has demonstrated a substantial improvement of pulmonary artery systolic pressure in pulmonary hypertensive leukaemic patient treated with tipifarnib.^15^

Our study addresses for the first time the effect of farnesyltransferase inhibition in prevention of chronic hypoxia-induced pulmonary hypertension and in the regulation of pulmonary endothelial function *in vitro* and *in vivo*.

## 2. Methods

An expanded Methods section is provided in the Supplementary data, see Supplementary material online.

### 2.1 Chronic hypoxia-induced pulmonary hypertension

#### 2.1.1 Normoxia and chronic hypoxia studies *in vivo*

All studies were conducted in accordance with UK Home Office Animals (Scientific Procedures) Act 1986 and institutional guidelines with the Imperial College ethics review board approval. 10–12 weeks old C57BL male mice (20 g; Charles River, Margate, UK) were either housed in normal air or were placed in a normobaric hypoxic chamber (FIO2 10%) for 2 weeks (*n* =  6–8/group). Mice were treated twice daily by oral gavage with either vehicle (2% carboxymethylcellulose in PBS) or tipifarnib (100 mg/kg/body weight).Treatments started one day prior hypoxia exposure. Development of PH was verified as described previously.^4^ At 2 weeks, the animals were weighed and anesthetized (Hypnorm 0.25 mL/kg; Midazolam 25 mg/kg IP), and right ventricular systolic pressure (RVSP) was measured in a closed chest via direct cardiac puncture in the spontaneously breathing, anesthetized animal. A 27G needle, connected to a blood pressure transducer (MLT0670, ADInstruments) and to PowerLab Data Acquisition system (ADInstruments), was introduced within the second intercostal space proximal to the sternal angle with an inclination of 45 degrees clockwise. All data were then analysed using LabChart 7 software. The animals were then euthanized, the hearts were removed, and the individual ventricular chambers were weighed. Right ventricular hypertrophy was assessed as the ratio of right ventricle/left ventricle + septum. The right lungs were snap-frozen in liquid nitrogen and stored at −80 °C for biochemical measurements. The left lungs were fixed by inflation with 10% formalin, embedded in paraffin, and sectioned for histology. Transverse lung sections were stained with van Gieson’s elastic method or smooth muscle-actin antibody (Sigma) and counterstained with Gill’s No. 2 haematoxylin (Sigma, GHS216). Vascular muscularization was defined as the proportion of vessels (<50 µm diameter) with immunoreactivity for smooth muscle actin (as evidence for muscularization) over the total number of vessels stained with elastin.

### 2.2 PAH lung sections for immunofluorescent staining of farnesylated proteins

Archived lung tissue sections from patients undergoing transplantation for PAH and relevant control material was obtained from the Papworth Hospital Cambridge and Hammersmith Hospital London tissue banks with local ethics committee approval (REC reference number 2001/6003 for Hammersmith Hospital and 08/H0304/56 + 5 for Papworth Hospital) and informed written consent. The investigation conformed to the principles outlined in the Declaration of Helsinki.

### 2.3 Immunostaining of lung sections

Lung sections were immunostained using a rabbit anti-farnesyl-proteins antibody (Milipore, AB4073) and a mouse anti-alpha-smooth muscle actin antibody (Dako, M0851), and fluorescently-labelled secondary antibodies. Images were taken under the confocal laser scanning fluorescence microscope (Leica TCS SP5) and analysed with Image J.

### 2.4 Cell culture and treatments

Human pulmonary artery endothelial cells (HPAEC; Promocell, C-12241, lot 7121003.5 and 4091902) and smooth muscle cells (HPASMCs; Promocell, C-12521, lot 9033101.6) were cultured, as described in Ref. 4. The cells were used between passages 3–6. The cells were kept under normoxia or were exposed to hypoxia (2% O_2_) for 2–72 h. In experiments involving a short-term (2 h) exposure to hypoxia, tipifarnib (Selleckchem) was added to the cells 1 h before the hypoxic exposure at 0.1 µmol/L. In experiments involving a more prolonged exposure to hypoxia (24–72 h), tipifarnib was added to the cells at the start of hypoxic exposure.^4^

#### 2.4.1 Intracellular calcium levels

HPAECs grown in 96-well optical bottom plates (Costar, 3603) were left untreated or were treated with 0.1 µmol/L tipifarnib for 24 h. Calcium flux was induced by thrombin (Sigma, T4648, 1 U/mL) or ionomycin (Sigma, I3909, 1 µmol/L) for 3 min. Intracellular calcium levels were studied with Fluo4 NW Calcium Assay kit (Invitrogen, F36206), according to the manufacturer’s protocol. Images of cells were taken under the confocal laser scanning fluorescence microscope (Leica TCS SP5) and the intensity of Fluo4 fluorescence (excitation 494 nm and emission 516 nm) was measured with Image J.

### 2.5 Isometric wire myography

Myography was performed on pulmonary artery segments isolated from control C57BL6 mice or mice exposed to chronic hypoxia. Intralobar pulmonary artery was removed, dissected free of fat and connective tissues and the vessel segments placed in Mulvany wire myographs to measure contraction and relaxation responses. Throughout the experiment, tissues were immersed in physiological salt solution (PSS, NaCl 1.18 x 10^−^^1^ mol/L, KCl 4.7 x 10^−^^3^ M, MgSO_4_ 1.17 x 10^−^^3^ mol/L, CaCl_2_ 2.5 x 10^−^^3^ mol/L, KH_2_PO_4_ 1.0 x 10^−^^3^ mol/L, EDTA 2.7 x 10^−^^5^ mol/L, and glucose 5.5 x 10^−^^3^ mol/L) at 36 °C, bubbled with 95% O_2_ and 5% CO_2_. The tension of the vessel was normalized to a tension equivalent to that generated at 90% of the diameter of the vessel at 100 mmHg. Changes in arterial tone were recorded via a PowerLab/800 recording unit (ADI instruments Pty Ltd., Australia), and analysed using Chart 6.0 acquisition system (ADI instruments). Arteries were exposed to three challenges of high potassium solution (KPSS; same composition as PSS except that NaCl is replaced with KCl 1.24 x 10^−^^1^ mol/L) followed by a washout period of 10 min. Concentration response curves to 9,11-dideoxy-11α,9α epoxymethanoprostaglandin F2α (U46619; 10^−^^9^–3.10^−^^7^ mol/L) and phenylephrine (10^−^^8^–10^−^^4^ mol/L) were performed on each of the vessels. Dilatory response curves were recorded in pulmonary arteries pre-contracted with an EC80 concentration of U46619, and vasodilator responses to either acetylcholine (10^−^^8^–10^−^^4^ mol/L) or SNP response curve (10^−^^8^–3.10^−^^5^ mol/L) were assessed. Force was recorded via a PowerLab/800 (AD Instruments Ltd., UK) and analysed using Chart 6.0 acquisition system (AD Instruments Ltd., UK).

### 2.6 Proliferation, metabolic activity, and apoptosis assays

HPAECs and HPASMCs proliferation was evaluated using bromodeoxyuridine (BrdU) assay (Millipore), while cell viability and caspase activation were assessed using the kit ApoTox-Glo™ (Promega). Metabolic activity (NADH/NADPH levels) was measured in CellTiter 96^®^ Aqueous One Solution Cell Proliferation Assay System (Promega, USA). The levels of cleaved caspase 3 in tipifarnib-treated cells and mouse lungs were analysed by western blotting. Apoptotic DNA fragmentation was studied with TACS 2 TdT-DAB *In Situ* Apoptosis Detection Kit (Trevigen, 4810-30K).

### 2.7 Endothelial permeability

Endothelial permeability was measured in Transwell assay.^4^

### 2.8 Rho GTPases protein expression and activity

Active (GTP-bound) RhoA, RhoB levels were measured in pulldown assays.^4^ Ras GTP-binding was studied with Raf-1–GST fusion construct encoding residues 51–131 of the human Raf-1 RBD (generously provided by Johannes L. Bos, Molecular Cancer Research, UMC Utrecht, The Netherlands).^16^

### 2.9 Activity measurement of farnesyltransferase and geranylgeranyltransferase

The protocol was adapted from Berndt et al.,^17^ replacing the FPP/[H^3^]GGPP and peptide substrates with non-radioactive FPP/GGPP and fluorophore-conjugated peptides. The prenylation substrates, nitrobenzofurazan (NBD)-GCVLS (FTase substrate), and NBD-GCVLL (GGTase-1 substrate) become strongly fluorescent upon prenylation.

### 2.10 Globular/filamentous actin quantification

The ratio of globular (G) actin to filamentous (F) actin was measured with G/F-actin In vivo Assay Kit (Cytoskeleton, Denver, CO, USA).

### 2.11 Real-time RT-PCR analysis

First-strand cDNAs were synthesized directly from HPAECs using the Superscript Cells Direct cDNA Synthesis Kit (Invitrogen) according to the manufacturer’s protocol. Polymerase chain reactions (PCRs) were performed using specific primer pairs for NOS3 (Hs01274659_m1) and GAPDH (Hs02758991_g1), from Taqman^®^.

### 2.12 RhoB mutant overexpression

Overexpression of AdGFP (adenoviral control), farnesylated-only RhoB (F-RhoB) and geranylgeranylated-only RhoB (GG-RhoB), was induced by adenoviral gene transfer.^4^ The last four C-terminal aminoacids of the F-RhoB were CLVS. In GG-RhoB, 16 C-terminal residues of RhoB were replaced by 13 C-terminal residues of RhoA, with the last four residues CLVL.^18^ CMV3/zeo HA-F-RhoB and CMV/zeo HA-GG-RhoB were a kind gift of Professor George Prendergast, Lancaster Avenue, Wynnewood, PA). The adenoviral vectors for RhoB mutant overexpression were from Welgen, Worcester, MA, USA).

### 2.13 Label- free quantitative proteomics

Cells lysates were separated by SDS-PAGE. After in-gel digestion of the separated proteins, tryptic peptides were analysed by LC-MS/MS. Data analysis was performed by applying various criteria to ensure certainty in the assignment of protein identifications and to select those that were differentially expressed. Functional associations of the identified proteins were analysed with STRING (Search Tool for the Retrieval of Interacting Genes/Proteins 10.0) database (http://string-db.org/, 10 January 2017) and Ingenuity Pathway Analysis version 01-07.

### 2.14 Statistical analysis

Statistical analysis was performed using GraphPad Prism 6 Software. Comparisons were carried out with unpaired Student’s *t-*test or ANOVA with Dunnett’s, Tukey or Bonferroni post-hoc tests, as appropriate. Statistical significance was accepted for *P *<* *0.05. Data are expressed as means ± SEM.

## 3. Results

### 3.1 Tipifarnib prevents development of chronic hypoxia-induced pulmonary hypertension in mice

**Figure 1:**
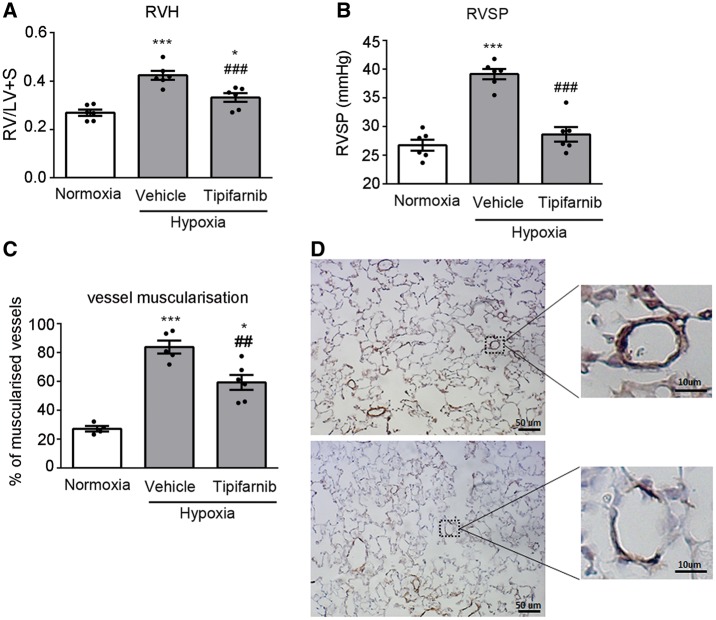
Tipifarnib attenuates development of chronic hypoxia-induced pulmonary hypertension. (*A*) right ventricular hypertrophy (RVH), (*B*) Right ventricular systolic pressure (RVSP), (*C, D*) vessel muscularization in the lungs of normoxic and chronically hypoxic mice treated with vehicle or tipifarnib (100 mg/kg). In (*D*) top panel shows smooth muscle actin (SMA) staining in lung sections from hypoxic control lungs and bottom panel shows SMA staining in lungs from tipifarnib-treated hypoxic mice. Magnified boxed areas illustrate changes in muscularization of small intrapulmonary arteries. In (*A–C*) values are means ± SEM of *n* = 6; *P < 0.05, **P < 0.01, ***P < 0.001 vs. normoxic control; ^##^*P* < 0.01, ^###^*P* < 0.001 vs. hypoxic control. 1-way ANOVA with Tukey post-test.

Mice exposed to chronic hypoxia for 2 weeks developed pulmonary hypertension, characterized by an increase in the right ventricular systolic pressure (RVSP), right ventricular hypertrophy (RVH) and muscularization of small (<50 µm diameter) intrapulmonary arteries (*Figure 1A–D*). Pulmonary vascular muscularization was accompanied by an increase in the protein expression of cell proliferation marker PCNA in the lung (see Supplementary material online, *Figure S1*).

Following oral treatment with tipifarnib (100 mg/kg, twice daily), the mice appeared healthy and no treatment-related mortality or significant weight loss were noted during the experiment (see Supplementary material online, *Figure S2*). Twice-daily oral administration of tipifarnib at 300 mg/kg was shown to alleviate symptoms of PH in a leukaemic patient.^15^ However, in mice a lower dose of 100 mg/kg twice daily efficiently inhibited growth of H-ras transfected tumours and therefore this dose was chosen in our investigation.^19^ Tipifarnib prevented the hypoxia-induced increase in RVSP and significantly reduced RVH and pulmonary arterial muscularization in hypoxic mice (*Figure 1A–D*). The effects of tipifarnib were accompanied by a significant reduction in the lung expression of PCNA (see Supplementary material online, *Figure S1*).

### 3.2 Tipifarnib reduces protein farnesylation in the lung

**Figure 2:**
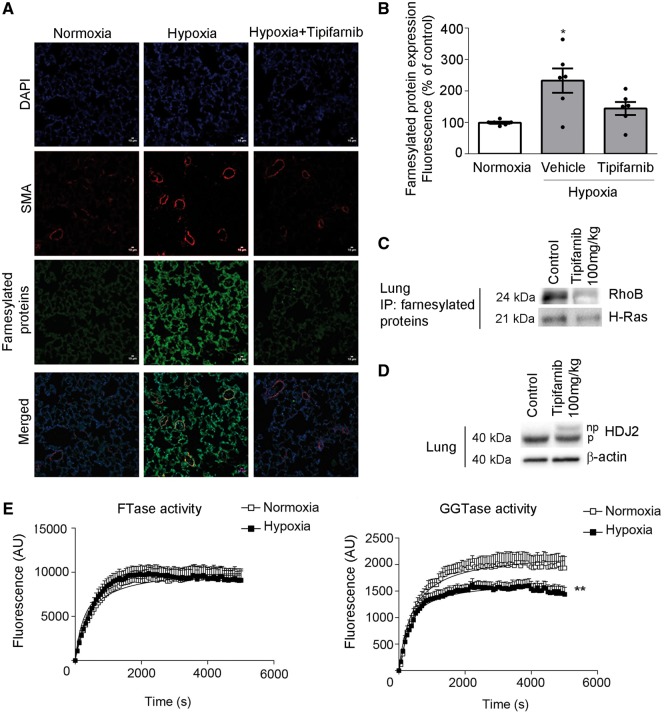
Tipifarnib reduces protein farnesylation in lungs of hypoxic PH mice. (*A*) Immunofluorescent images of farnesylated proteins and α-smooth muscle actin in the lungs of normoxic mice and vehicle- and tipifarnib-treated hypoxic mice, as indicated. Bar = 10µm. (*B*) Changes in protein farnesylation in mouse lungs. Values are mean fluorescence intensities ±SEM of *n* = 6/group; *P < 0.05. 1-way ANOVA with Tukey post-test. (*C, D*) Representative western blots showing tipifarnib-induced accumulation of unprenylated (np; upper band) form of farnesylation marker protein, HDJ2 and reduction in the levels of farnesylated RhoB and H-Ras in the mouse lung. (*E*) FTase activity and GGTase activity in the lungs of normoxic (clear boxes) and chronically hypoxic mice (black boxes). Each data point represents a mean± SD of *n* = 5–6. The V_max_ and K_m_ of the curves were compared using the Michaelis–Menten equation fitted to data points. **P < 0.005, comparison with normoxic controls, for both V_max_ and K_m_.

Hypoxia increased protein farnesylation in the lung, while tipifarnib had an inhibitory effect (*Figure 2A and B*). The effectiveness of the selected dose of tipifarnib was documented by an increase in the levels of unprenylated farnesylation marker protein HDJ2 and reduction in the levels of farnesylated RhoB and H-Ras in mouse lungs (*Figure 2C and D* and see Supplementary material online, *Figure S3*). The selectiveness of the antibody used to visualize farnesylated proteins was verified by western blotting (see Supplementary material online, *Figure S4*). To further investigate, whether protein farnesylation increases in PH lung, we examined the levels of protein farnesylation in rat lungs with monocrotaline (MCT)-induced PH and lungs of human patients with severe PAH. Consistent with the observations in the remodelled mouse lungs, MCT rat, and human PAH lungs showed increased levels of protein farnesylation, compared with healthy controls (1.6- and 1.5-fold increase respectively, *P* < 0.05) (see Supplementary material online, *Figures S5* and *S6*).

Hypoxic mouse lungs did not show changes in protein levels of FTase, GGTase or the activity of FTase, but the activity of GGTase was significantly reduced compared with normoxic controls (*Figure* 2E and see Supplementary material online, *Figure S7*). Reduction in GGTase activity may potentially enhance protein farnesylation by increasing bioavailability of isoprenoid intermediates and/or protein substrates utilized by FTase.^20^

### 3.3 Tipifarnib increases endothelium-dependent relaxation of pulmonary arteries

**Figure 3:**
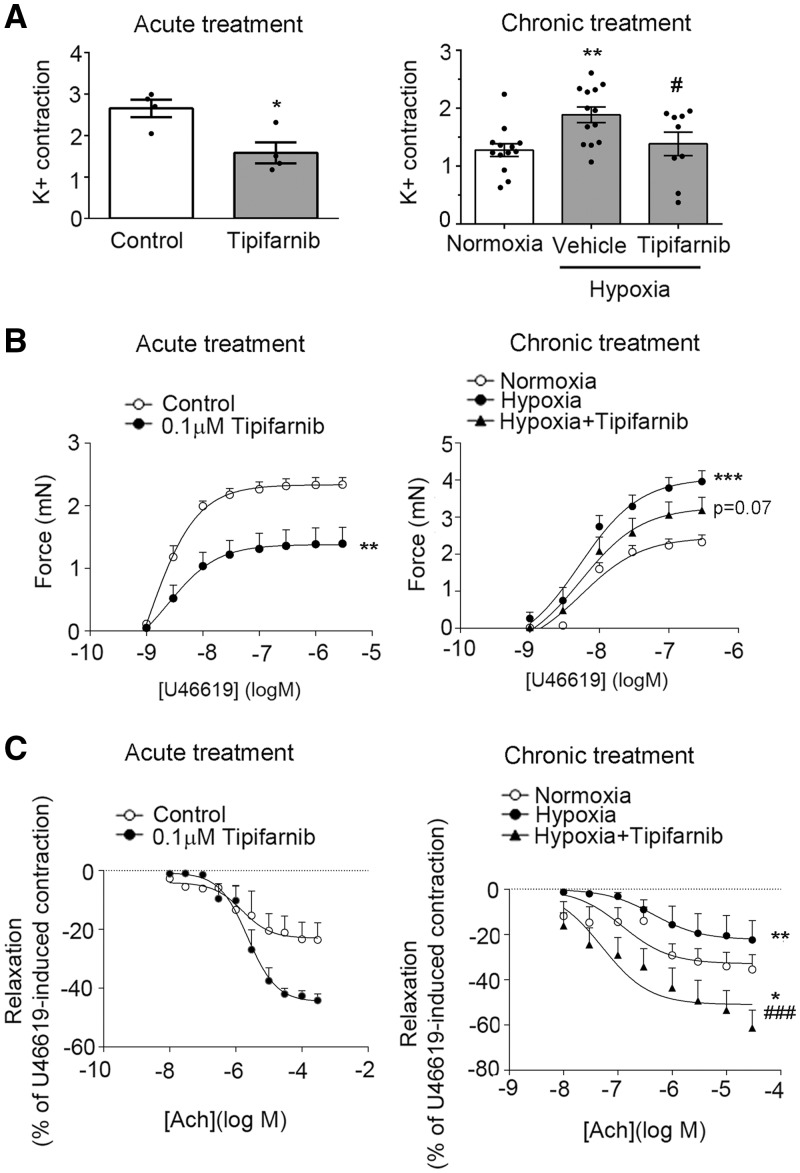
Tipifarnib increases endothelium-dependent relaxation of pulmonary arteries. In (*A–C*) graphs on the left show reactivity of intrapulmonary arteries pretreated for 2 h with 0.1 μmol/L tipifarnib (Acute treatment), while graphs on the right show reactivity of intrapulmonary arteries isolated from normoxic, hypoxic and hypoxic tipifarnib-treated mice (Chronic treatment). (*A*) Contraction to high potassium solution; (*B*) contraction to U44162; (*C*) relaxant response to acetylcholine. Values are means ± SEM of *n* = 4 for acute treatment and *n* = 8–12 for chronic treatment. 0% relaxation corresponding to the level of precontraction induced by 3 x 10 ^−^^6^ mol/L U44162. Statistical significance was determined using a (*A*) unpaired *t*-test or (*B, C*) a two-way ANOVA with repeated measures and a Bonferroni post-hoc test. *P < 0.05, **P < 0.01, ***P < 0.001 vs*.* control; ^#^*P* < 0.05, ^###^*P* < 0.001 vs*.* hypoxic control.

We evaluated the effect of tipifarnib on vascular reactivity of mouse intrapulmonary arteries. Acute (2h) *ex vivo* treatment with tipifarnib decreased contraction of intrapulmonary arteries to high K^+ ^and prostaglandin H2 analogue, U46619 and increased acetylcholine-mediated vasorelaxation (*Figure 3A–C*). Tipifarnib-induced increase in vasorelaxation was prevented by NOS inhibitor L-NAME, indicative of increased endothelial NOS signaling (see Supplementary material online, *Figure S8*). Acute tipifarnib slightly reduced SNP-induced vasorelaxation (see Supplementary material online, *Figure S9*).

Chronic hypoxia induced pulmonary arteries hypercontractility in response to high K^+ ^and U46619 and decreased vasorelaxation to acetylcholine (*Figure 3A–C*). Chronic (2 weeks) treatment with tipifarnib reduced K^+^- and U46619-induced contractility and significantly improved acetylcholine-induced vasorelaxation of intrapulmonary arteries from hypoxic lungs (*Figure 3A–C*) but had little effect on relaxation to SNP (see Supplementary material online, *Figure S9*). Twenty-four-hour treatment with tipifarnib did not significantly affect intracellular calcium levels in the untreated or thrombin-treated HPAECs (see Supplementary material online, *Figure S10*).

### 3.4 Tipifarnib reduces hypoxia-induced HPAEC and HPASMC proliferation and inhibits the activities of H-Ras and small GTPases *in vitro* and *in vivo*

**Figure 4:**
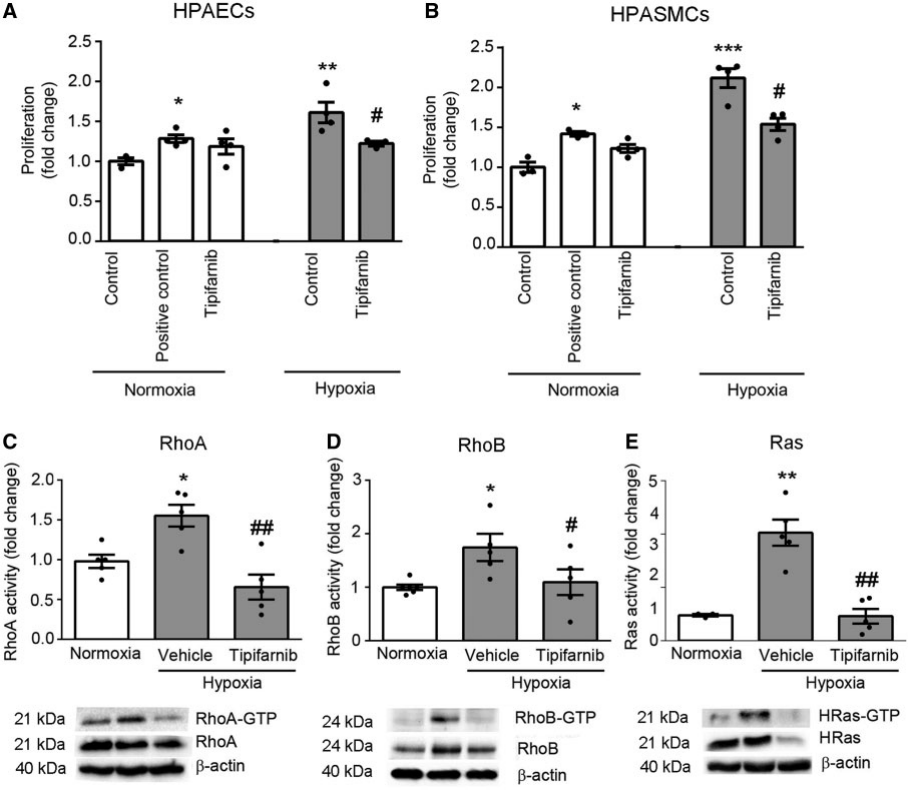
Tipifarnib reduces cell proliferation and small GTPases activity. Proliferation of (*A*) HPAECs or (*B*) HPASMCs cultured in media containing 1% FBS in normoxia or in hypoxia with or without tipifarnib for 72 h (0.1 µmol/L; BrdU assay). Cells in positive controls were stimulated with 10% FBS. Activities of (*C*) RhoA, (*D*) RhoB and (*E*) Ras were assessed in lungs of mice exposed to normoxia or chronic hypoxia and treated with either vehicle or tipifarnib, as indicated. β-actin was used as a normalization control. Data represent mean fold- change ±SEM of *n* = 5. *P < 0.05, **P < 0.01 vs. normoxic control; ^#^*P* < 0.05, ^##^*P* < 0.01 vs. hypoxic control. 1-way ANOVA with Tukey post-test.

Tipifarnib inhibited hypoxia-induced HPAEC and HPASMC proliferation (*Figure 4A and B*), without affecting cell viability or apoptosis *in vitro* or *in vivo* (see Supplementary material online, *Figure S11*). The drug also prevented hypoxia-induced activation of Ras and Rho proteins in the lung, without altering protein expression (*Figure 4C–E* and see Supplementary material online, *Figure S12*). In cultured cells, reduction in RhoB activity was noted at 2 h and RhoA and Ras activity at 24 h of treatment with tipifarnib (see Supplementary material online, *Figure S13*).

### 3.5 Tipifarnib reduces F-actin levels and stabilizes eNOS mRNA in pulmonary endothelial and smooth muscle cells

Rho-induced actin polymerization reduces the stability of eNOS mRNA and decreases NO production.^21^ To better understand the mechanism of vasorelaxing actions of tipifarnib, we studied the effect of the drug on actin polymerization in cultured HPAECs and HPASMCs.

**Figure 5:**
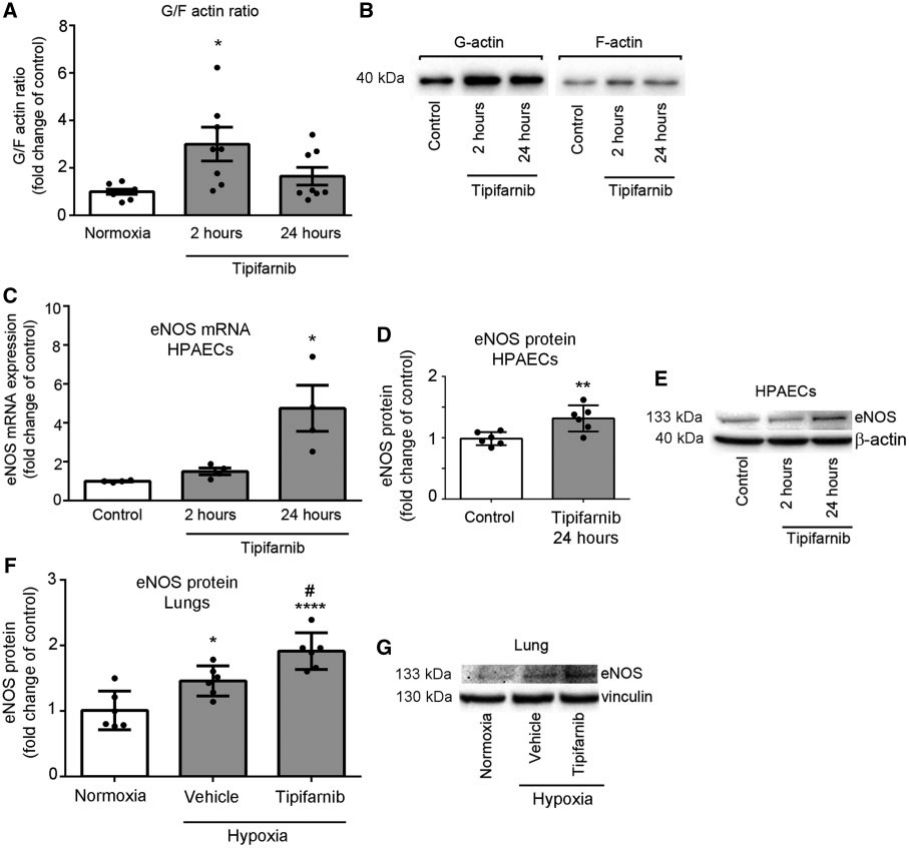
Tipifarnib depolymerizes actin and increases eNOS expression in HPAECs and mouse lungs. (*A*) Graph and (*B*) representative western blot show changes in globular/filamentous (G/F) actin ratio in HPAECs treated with tipifarnib (0.1 µmol/L) for 2–24 h, as indicated. *n* = 7–8. (*C*) eNOS mRNA and (*D, E*) eNOS protein expression in cells stimulated with tipifarnib; *n* = 5–6. (*F, G*) eNOS protein expression in the lungs of mice exposed to normoxia or chronic hypoxia and treated with either vehicle or tipifarnib, *n* = 6. Results are expressed as fold- change of controls; Data represent mean ± SEM *P < 0.05 vs. normoxic control. 1-way ANOVA with Tukey post-test.

Tipifarnib reduced the levels of polymerized actin (F-actin) in hypoxic HPAECs and HPASMCs and prevented the localization of phosphorylated myosin light chain (p-MLC) to stress fibres, indicative of reduced actomyosin contractility (*Figure 5A and B* and see Supplementary material online, *Figure S14 A* and *B*).

Increase in the levels of unpolymerized, globular (G)-actin correlated with increased gene and protein expression of eNOS in tipifarnib-treated HPAECs and mouse lungs (*Figure 5A–G*).

### 3.6 Farnesylated RhoB induces an activated, pro-proliferative cell phenotype

**Figure 6:**
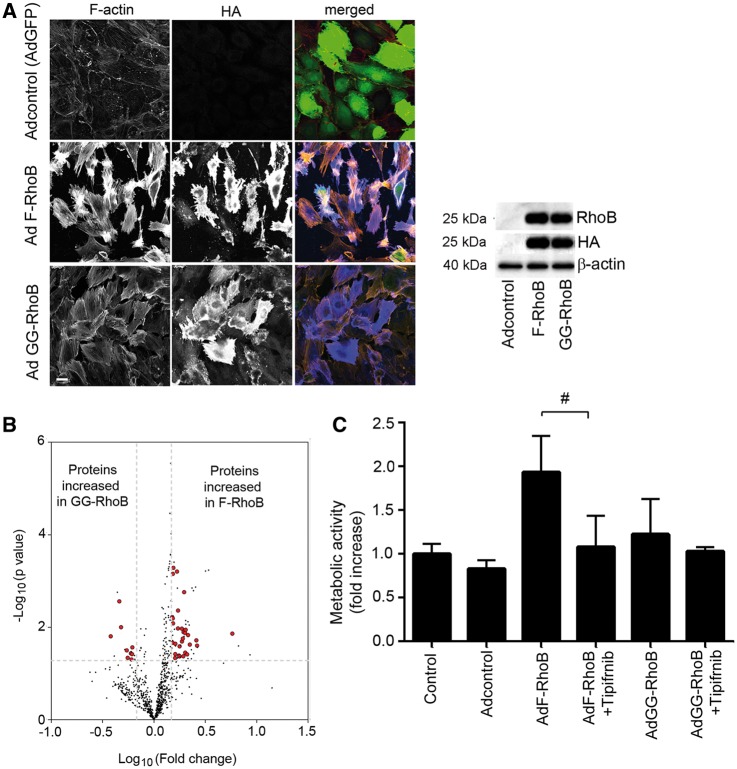
Farnesylated RhoB induces stress fibre formation and increases metabolic activity in HPAECs. (*A*) The effect of 24 h-overexpression of AdF-RhoB and AdGG-RhoB on the endothelial actin cytoskeleton. The RhoB mutants were HA-tagged and co-expressed with GFP and AdGFP was used as adenoviral control (Adcontrol). In merged images F-actin is red, HA is blue and GFP is green. Bar = 10 µm. Western blot shows equal RhoB expression levels in cells infected with Ad F-RhoB and Ad GG-RhoB. (*B*) Volcano plot illustrates differences in protein expression profile in cells overexpressing GG-RhoB or F-RhoB. Dots in the upper right hand and the left hand corner represent significantly altered expression, with the enlarged red dots marking proteins that affect cell metabolic activity/biosynthesis. All up- or down- regulated proteins are listed in the, see Supplementary material online, *Table S1* and those that are involved in metabolic activity/biosynthesis are indicated. The broken lines indicate thresholds of 1.5-fold-change and *P*-value 0.05; *n* = 5. (*C*) Changes in the metabolic activity in cells overexpressing AdGFP, AdF-RhoB and AdGG-RhoB and treated with tipifarnib, as indicated. Values are means ± SEM of *n* = 5. **P < 0.001 vs. control, ^#^*P* < 0.05 comparison, as indicated. 1-way ANOVA with Tukey post-test.

To evaluate the effect of changes in RhoB prenylation on pulmonary endothelial cell phenotype, we overexpressed farnesylated-only RhoB (F-RhoB) or geranylgeranylated-only RhoB (GG-RhoB) in HPAECs. Both mutants induced stress fibre formation and cell rounding in HPAECs within 10–24 h of overexpression, with F-RhoB having a more pronounced effect (*Figure* 6). Differential effects of the two mutants were more clearly seen at earlier timepoints of mutant overexpression (6 h), when F-RhoB, but not GG-RhoB significantly increased endothelial permeability (see Supplementary material online, *Figure S15*).

F-RhoB also increased metabolic/proliferative activity in HPAECs and its effects were inhibited by tipifarnib (*Figure* 6*C*).

**Figure 7:**
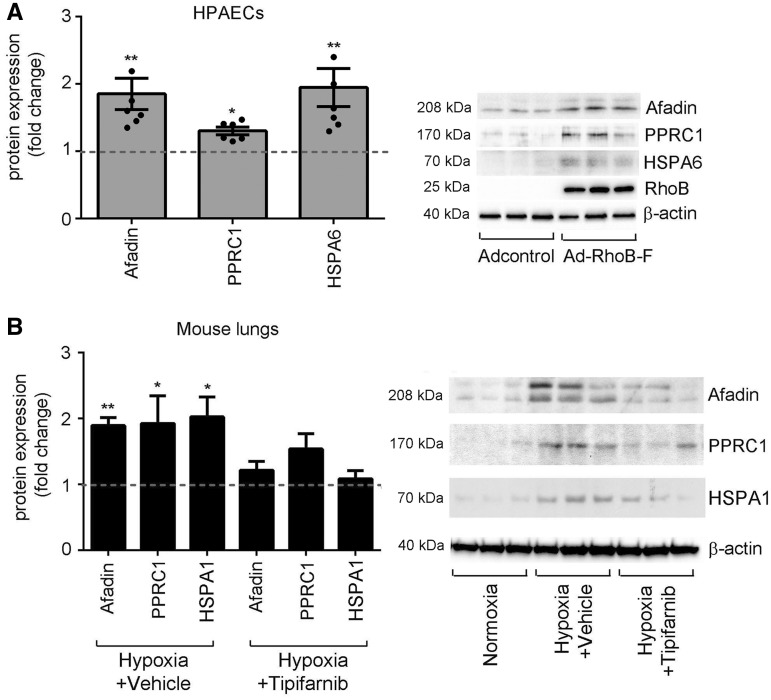
Farnesylated RhoB increases expression of proteins regulating cell survival, activation of cell cycle and mitochondrial biogenesis. (*A*) Fold-change in protein levels of afadin, PPRC1 and HSPA6 in HPAECs overexpressing F-RhoB. Bars are means ± SEM, *P < 0.05; **P < 0.01, comparison with adenoviral controls (Adcontrol); *n* = 6. Representative western blots are shown beside the graph. (*B*) Fold-change in protein levels of afadin, PPRC1 and HSPA1 in normoxic mouse lungs (Normoxia), hypoxic mouse lungs (Hypoxia + Vehicle) and lungs of hypoxic mice treated with Tipifarnib (Hypoxia + Tipifarnib). HSPA6 is not expressed in rodents and therefore the expression of HSPA1, belonging to the same HSP70 family of proteins and regulated by F-RhoB, was analysed. Values are means ± SEM of *n* = 6 and the broken line indicates control level. *P < 0.05; **P < 0.01, comparison with normoxic controls. 1-way ANOVA with Tukey post-test. Representative western blots are shown beside the graph.

Label-free proteomics analysis showed significantly increased expression of 60 proteins in cells overexpressing F-RhoB and 16 proteins in cells overexpressing GG-RhoB (*Figure* 6*B* and see Supplementary material online, *Table S1*). Pathway analysis linked F-RhoB with activation of cell growth and biogenesis, while GG-RhoB with cell cycle inhibition and protein ubiquitination (see Supplementary material online, *Tables S1* and *S2*). Consistent with the postulated role of RhoB, we observed a significant increase in the levels of F-RhoB-regulated proteins, such as afadin (cell survival, PDGF and Akt signalling, angiogenesis),^22^^,^^23^ heat shock 70 proteins^24^ and peroxisome proliferator-activated receptor gamma coactivator- related 1(PPRC1; transcription factor of mitochondrial biogenesis),^25^ in hypoxic mouse lungs, while treatment with tipifarnib had an inhibitory effect (*Figure* 7).

Consistent with the results of proteomic profiling, F-RhoB also increased BrdU incorporation in HPAECs and platelet-derived growth factor-BB (PDGF-BB) -stimulated HPASMCs while GG-RhoB had an inhibitory effect (see Supplementary material online, *Figure S16*).

### 3.7 Differential effect of Tipifarnib and simvastatin on protein expression of small GTPases

Statin treatment carries health risks associated with increased accumulation of unprenylated Ras proteins contributing to their sudden activation upon drug withdrawal.^26^ To highlight potential benefits of FTI-based therapy, we compared changes in protein expressions of RhoA, RhoB, and Ras in cells treated with tipifarnib and simvastatin. Tipifarnib did not significantly alter protein expressions of Ras, RhoA or RhoB, in contrast to simvastatin, which markedly upregulated the expressions of all studied proteins (see Supplementary material online, *Figure S17 A*–*C*). This differential effect may result from the fact that FTIs selectively inhibit protein farnesylation, preserving, or even increasing protein geranylgeranylation, important for the ubiquitination and subsequent degradation of Ras proteins. In support of this hypothesis, we observed that co-incubation of tipifarnib-treated cells with GGTase-1 inhibitor, GGTI-298 substantially elevated protein expressions of RhoA and H-Ras to the levels induced by simvastatin (see Supplementary material online, *Figure S17*).

## 4. Discussion

In this study, we show that oral administration of non-peptidomimetic farnesyltransferase inhibitor tipifarnib to chronically hypoxic mice, significantly improves eNOS-dependent vasodilatation, reduces pulmonary vascular remodelling and prevents hypoxia-induced right heart hypertrophy. FTIs have shown promise in treatment of vascular pathologies associated with atherosclerosis,^27^ progeria^28^ and systemic hypertension^29^ and here we document potential role of FTIs in treatment of pulmonary hypertension.

**Figure 8:**
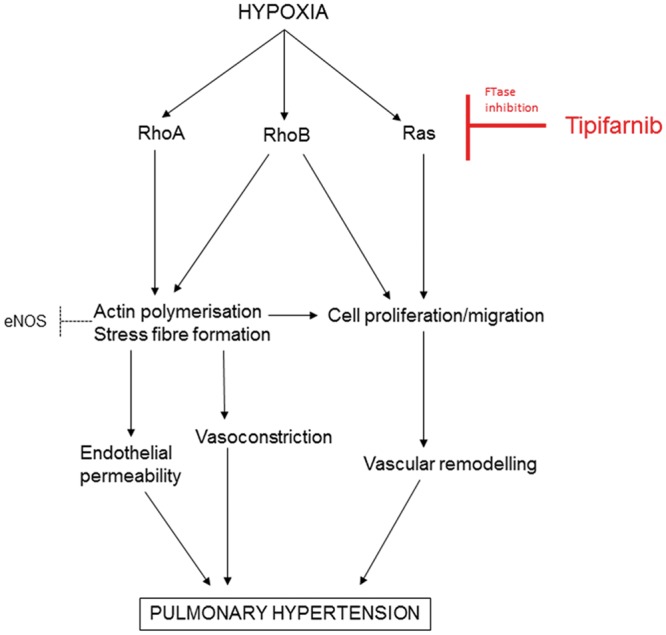
A schematic diagram of the proposed signaling pathway. FTase: farnesyltransferase; ROCK: Rho kinase; mDia: mammalian homolog of Drosophila diaphanous.

Reduction in pulmonary vascular remodelling by tipifarnib may be attributed, at least in part, to the inhibition of expression and activity of Ras proteins (*Figure* 8). Inhibition of Ras farnesylation inhibits neointimal thickening of coronary artery,^30^ reduces PDGF-induced VSMC proliferation and inhibits the actions of Ras effectors such as endothelin-1 (ET-1), or TGF-β.^31^ RhoB is a downstream effector of Ras and regulates intracellular trafficking of EGF and PDGF receptors and kinases src and Akt, enhancing cell proliferation and survival.^32^^,^^33^^,^^34^ Several human and animal studies have also established the importance of RhoA and its downstream mediator, ROCK, in pulmonary arterial contractility and remodelling.^34^ Interestingly, tipifarnib reduced the activity of RhoA which, unlike Ras or RhoB, can only be geranylgeranylated. This effect may result from increased NO/cGMP/PKG-mediated phosphorylation of RhoA, known to promote arterial vasodilation.^35^

Tipifarnib decreased pulmonary arterial vasoconstriction to high K^+ ^and U46619 in a NOS-independent manner. Considering that RhoB has a short half-life (∼2 h for RhoB compared to ∼24–31 h for Ras and RhoA) and that the acute treatment with tipifarnib decreased RhoB activity without affecting RhoA or Ras, this response can be attributed to the inhibition of RhoB.

Inhibition of Rho signalling can also account for NOS-dependent increase in vasorelaxation seen in pulmonary arteries treated with tipifarnib. Rho/Rho kinase inhibitors increase the levels of unpolymerized, globular (G)-actin, known to stimulate eNOS expression either by binding to the enhancer region of eNOS promoter^36^ and/or by stabilizing eNOS mRNA.^21^ In addition, G-actin can bind to eNOS residues 326-333 and shift its enzymatic activity from superoxide formation toward NO production,^37^ or stimulate eNOS activity via PI3-kinase/Akt-mediated phosphorylation.^38^ Further, depolymerization of actin has been shown to activate eNOS by reducing stiffness of the cell cortex.^39^

Accordingly, we observed that increased eNOS gene and protein expression co-incided with actin depolymerization and reduced activities of RhoA and RhoB. Tipifarnib improved acetylcholine-induced vasorelaxation of hypoxic intrapulmonary arteries and increased eNOS protein expression in the lung. Interestingly, total RhoB knockout in mice did not affect pulmonary vasoreactivity, possibly as a result of a compensatory increase in RhoA expression,^4^ not seen in tipifarnib-treated lungs. Tipifarnib did not alter intracellular Ca^2+ ^levels in the untreated or thrombin-treated cells, suggesting that modulation of Rho rather than Ca^2+^-calmodulin signalling^40^ contributed to the vasorelaxing effects of the drug.

Tipifarnib is the most clinically relevant agent available, with proven on-target effects *in vivo.*^41^ Clinical trials showed that the drug is relatively well tolerated, and does not significantly affect cardiovascular function.^42^^,^^43^ It is important to note, that the outcomes of anti-farnesylation therapies may vary, depending on disease mechanisms, experimental design, dose and route of drug administration, FTI specificity and the mode of action. In this study, tipifarnib reduced pulmonary arterial remodelling and improved endothelium-dependent vasorelaxation in hypoxic mice in a mechanism involving inhibition of Ras and Rho signaling. However, in a mouse model of progeria, tipifarnib prevented loss of arterial media by reducing farnesylation of progerin. Permanent farnesylation of progerin is thought to destabilize nuclear lamina in arterial VSMCs, making them susceptible to shear stress-induced damage.^28^ In another study, one-daily intravenous administration of structurally unrelated peptidomimetic farnesyltraneferase inhibitor, FTI-276 improved cardiac remodelling but failed to significantly alter endothelium-dependent vasorelaxation in spontaneously hypertensive rats.^29^

Data from our and other laboratories suggest that the equilibrium between protein farnesylation and geranylgeranylation is important in control of growth responses in the cardiovascular system.^44^^,^^45^ In particular, prenylation status of RhoB is likely to play a role. FTI-induced gain-of-function increase in GG-RhoB inhibits cancer cell proliferation^18^ and we have shown that GG-RhoB inhibits PDGF-induced HPASMC proliferation. In contrast, F-RhoB stimulates cell proliferation and increases expression of proteins important for cell survival, cell division, mitochondrial biogenesis, and energy metabolism. Increased expression of F-RhoB-regulated proteins was also observed in PH mouse lungs, providing an argument for the role of RhoB farnesylation in lung responses to chronic hypoxia. Mechanisms inducing differential prenylation of proteins such as RhoB and affecting the activity of prenyltransferases are poorly understood.^46^ It is possible that hypoxia-induced increase in protein farnesylation results from selective inhibition of GGTase activity^20^ or changes in expression of regulatory proteins.^46^

Identification of other FTI targets important in the pathogenesis of PAH, will require further studies. Database searches for potential farnesylation targets (proteins terminating in CAAX motifs) identified dozens of proteins, some of which can potentially be implicated in the pathogenesis of PH. These include several bone morphogenetic proteins, transforming growth factor-β precursors, serine/threonine kinase-11, and inositol-1,4,5-trisphosphate 5-phosphatases (I and IV), all of which are proteins with potential roles in the growth regulation.^47^

Our study highlights potential therapeutic benefits of selective inhibition of protein farnesylation, as opposed to the use of general prenylation inhibitors, such as statins. Statins are not effective in inhibiting protein farnesylation at safe therapeutic doses^9^ and induce the accumulation of unprenylated, immature forms of Ras proteins that maintain partial function.^7^ In contrast to simvastatin, tipifarnib did not increase protein expressions of Ras or Rho *in vitro* or *in vivo*, possibly as a result of alternative geranylgeranylation, targeting Rho proteins for proteasomal degradation.^7^ These effects were reversed by GGTi-995, supporting the argument that by shifting the balance towards protein geranylgeranylation, FTIs may exert better control over disease-related increases in Rho proteins expression and activity than statins.

Preliminary analysis of lung tissues from MCT rats and PAH patients showed increased protein farnesylation in the remodelled vessels and the surrounding tissues, suggesting a potential broader significance of this pathway in the pathogenesis of the disease. Such an assumption may appear too far-fetched, considering the complexity of vascular pathology in PAH.^48^ However, protein farnesylation increases in numerous other proliferative and inflammatory disorders^46^^,^^49^ and is likely to affect the function of a variety of cell types in addition to the cell types studied here. Hypoxia alone or in combination with other vascular stresses contributes to PAH pathology^1^ and RhoB can be activated by numerous other factors implicated in the pathogenesis of PAH, including tyrosine kinases TGF-β/bone morphogenetic protein/smad pathway and growth factors, fibroblast growth factor, epidermal growth factor, and PDGF.^3^^,^^33^ Further studies will need to address the role of FTIs in non-hypoxic models of PH and provide a detailed comparative analysis of the effects of FTIs and statins. Potential pro-apoptotic effects of FTIs will also need to be considered. Tipifarnib can induce apoptosis in malignant cells^50^ and the results of studies examining the effects of farnesyltransferase inhibition in normal adult tissues are unclear.^51^

To summarize, this study provides a molecular rationale for further exploring the family of farnesyltransferase inhibitors in treatment of pulmonary hypertension.

## Supplementary material

Supplementary material is available at *Cardiovascular Research* online.

## Supplementary Material

Supplementary Data
