# Supplementary material for: Tipifarnib prevents development of hypoxia-induced pulmonary hypertension
Source: Cardiovasc Res. 2017 Jan 5;113(3):276–87. doi: 10.1093/cvr/cvw258 (PMC5408956; doi:10.1093/cvr/cvw258)
Supplement: Supplementary Data [file cvw258_Supp.doc]

Tipifarnib prevents development of hypoxia-induced pulmonary hypertension.

Duluc et al.

**SUPPLEMENTAL MATERIAL**

Monocrotaline-induced PH rat lung

All studies were conducted in accordance with UK Home Office Animals (Scientific Procedures) Act 1986 and institutional guidelines with the Imperial College ethics review board approval. Lung sections of healthy and MCT adult male Sprague- Dawley rats (weight, 200–250 g; from Charles River, UK) were obtained from paraffin-embedded lungs from our previous study.^1^ Specifically, rats were injected with monocrotaline (MCT, 60 mg/kg; Sigma-Aldrich) subcutaneously and 4 weeks later mean pulmonary arterial pressure (mPAP) was measured by catheterization via right jugular vein (mPAP: MCT 45.1 ±9.1 mmHg vs control 18.5±3.6 mmHg, P<0.01), and the ratio of RV to LV plus the septum mass (RV/LV+septum) was used as an index of RV hypertrophy (MCT 0.46±0.11 vs control 0.24±0.01, P<0.001). Left lungs were inflated and fixed with 10% formalin for histology examination.

Cell culture. Human pulmonary artery endothelial cells (HPAECs) were grown on fibronectin-coated (bovine fibronectin, Sigma-Aldrich Company Ltd. Gillingham, Dorset, UK) plastic ware in Human Endothelial Cell Growth Medium 2 supplemented with 2% foetal calf serum and EGF (5 µg/L), basic FGF (10 µg/L), IGF (20 µg/L), VEGF (0.5 µg/L), ascorbic acid (1mg/L), heparin (22.5 mg/L) and hydrocortisone (0.5 mg/L). Human pulmonary artery smooth muscle (HPASMCs) were grown in the Human Smooth Muscle Cell Growth Medium 2 supplemented with 5% Foetal calf serum, EGF (0.5 µg/L) and FGF (2 µg/L). The cells were cultured under normal oxygen tension (20% O_2_, 5% CO_2_) or exposed to hypoxia (2% O_2_, 5% CO_2_, 92% N_2_) for 1- 48 hr. The cells and culture media were from PromoCell (Heidelberg, Germany). In experiments involving a short-term (2 hours) exposure to hypoxia, tipifarnib (Selleckchem) was added to the cells 1 hour before the hypoxic exposure at 0.1 µM.^2^ In experiments involving a more prolonged exposure to hypoxia (24 – 72 hours), tipifarnib was added to the cells at the start of hypoxic exposure. Following the treatment, the cells were used for studies of Rho GTPases expression, activity, F-actin organisation, cell proliferation and viability.

Endothelial Permeability.

Confluent HPAECs growing in Transwell filters (0.4µm pore size, VWR) were left untreated or were infected with AdGFP (Adcontrol), AdF-RhoB or AdGG-RhoB. 18 hours post-infection, fluorescent dextran (FITC-dextran, MW 40 kDa, Sigma; 0.5 g/L) was added to the top chamber of Transwell dishes for 1 hour and changes in endothelial barrier function were studied by the measurement of passage of FITC-dextran through the endothelial cell layer.

Immunofluorescence and confocal microscopy. The cells cultured on plastic coverslips (Nunc, cat no 174950) were fixed with 4% formaldehyde solution in PBS for 10 minutes at room temperature and permeabilised for 3 minutes with 0.1% Triton X-100. The cells were incubated in 2% bovine serum albumin (BSA, Sigma) in PBS for 45 minutes to block non-specific antibody binding and then incubated with mouse monoclonal anti-p-Ser20 MLC antibody (Sigma, 10 mg/L), washed 2x in PBS and incubated with FITC-labelled goat anti-mouse antibody (Jackson ImmunoResearch Laboratories, 115-095-062; 10 µg/ml) and 1mg/L of TRITC-phalloidin (Sigma) to stain actin filaments. Coverslips were washed 3x in PBS and mounted in Vectashield mountant containing nuclear stain DAPI.

To stain farnesylated proteins, dewaxed and rehydrated lung sections were subjected to heat-induced antigen retrieval in 10 mmol/L sodium citrate (pH 6.0) and 0.05% Tween 20, at 80°C for 20 minutes, and immunostained using the avidin-biotin-peroxidase complex (ABC Elite, Vector Laboratories) method and 3,3'-diaminobenzidine as a substrate. Sections were incubated with a rabbit anti-farnesyl-proteins antibody (Milipore, AB4073, 5µg/mL) and a mouse anti-alpha-smooth muscle actin antibody (Dako, M0851, 5µg/ml). Secondary antibodies used were Cy-5 anti-rabbit (Jackson ImmunoResearch Laboratories, 711-175-152, 5µg/ml) and TRITC anti-mouse secondary antibodies (Jackson ImmunoResearch Laboratories, 115-025-146, 5µg/ml). Sections incubated with purified non-immune rabbit IgG were used as staining controls. Slides were mounted in Vectashield mountant containing nuclear stain DAPI.

Images on cells were taken under the confocal laser scanning fluorescence microscope (Leica TCS SP5).

Western blot analysis. Following electrophoresis and protein transfer, the membranes were probed with the following primary antibodies: mouse monoclonal anti-RhoA (Santa Cruz Biotechnology; sc-418), rabbit anti-RhoB (Santa Cruz Biotechnology; sc-180), rabbit anti-PCNA (Santa Cruz Biotechnology; sc9857); rabbit polyclonal anti-HA (Santa Cruz Biotechnology; sc-805); mouse monoclonal anti-β-actin (Sigma; A2228); rabbit anti-eNOS (Millipore; 07-520), mouse anti-H-Ras (Millipore; MAB-3291), mouse anti- Ras (Millipore; 05-1072), rabbit anti- farnesyl-proteins (Millipore; AB4073), mouse anti-vinculin (Abcam; ab18058) and rabbit anti-HDJ2 (Abcam; ab3089), rabbit monoclonal anti-cleaved caspase-3 (Cell Signaling; 9664), rabbit monoclonal anti-Afadin (Thermoscientific, A7L9H48), rabbit anti-PPRC1 (Atlas Antibodies, HPA038511), rabbit anti-HSPA6 (Santa Cruz Biotechnology, sc-292204) and rabbit anti-HSPA1 (Santa Cruz Biotechnology, sc-133679). Secondary antibodies: goat anti-rabbit HRP-labelled (Sigma; AG154) and goat anti-mouse HRP-labelled antibody (Dako; 2016-07). Primary antibodies were used at 1mg/L and secondary antibodies at dilution 0.2 mg/L. The relative intensity of the immunoreactive bands was determined by densitometry using Image J software (Rasband, W.S., ImageJ, U. S. National Institutes of Health, Bethesda, Maryland, USA, http://imagej.nih.gov/ij/, 1997-2011). The results were normalised to β-actin or vinculin levels and expressed as fold- change over control. Untreated control samples were included in every experiment and all experiments were repeated at least 3 times (n≥3).

Immunoprecipitation of RhoB.

Confluent HPAECs grown in 3cm petri dishes were infected with adenoviruses to induce overexpression of F-RhoB or GG-RhoB. 24 hours post-infection, the cells were lysed in 1 mL lysis buffer containing 0.5% NP-40 (ThermoFisher Scientific), 10 mmol/L Tris-HCl pH 7.6, 150 mmol/L sodium chloride, 30 mmol/L sodium pyrophosphate, 5mmol/L EDTA with the protease and protein phosphatase inhibitor mix (ThermoFisher Scientific; 88266 and 78420). 50 µL of total cell lysates were reserved for subsequent analysis by electrophoresis and western blotting. The remaining cell lysates were pre-cleared by incubation with 30 µL of Protein G Sepharose (Sigma; P3296) for 1 hour. Sepharose beads were collected by centrifugation and the supernatants were incubated overnight with 5 µL of a rabbit anti-RhoB (Santa Cruz Biotechnology; sc-180) at 4°C. Then, 30 µL of Protein G Sepharose were added to the lysates for 1 hour and the beads were spun down in a benchtop centrifuge at 13 000 rpm for 3 minutes. The beads were washed 3 x in PBS, re-suspended in sample buffer, boiled and resolved by electrophoresis, followed by western blotting.

Semi-quantitative Reverse Transcriptase Polymerase Chain Reaction (RTPCR). To characterise changes in eNOS mRNA expression in HPAECs, total RNA was isolated using the RNeasy mini kit (Qiagen, Netherlands). Prior to cDNA synthesis, the RNA was treated with DNase I (Invitrogen) to remove any residual genomic DNA. 1 μg of total RNA was used for cDNA synthesis. First-strand cDNA synthesis was performed using Superscript II reverse transcriptase (Invitrogen). RT–PCR was performed with isoform-specific primers for NOS3 (Hs01274659_m1) and GAPDH (Hs02758991_g1), from Taqman®.

Adenoviral overexpression of RhoB prenylation mutants. Overexpression of farnesylated-only (F-RhoB) and geranylgeranylated-only (GG-RhoB) RhoB was induced by adenoviral gene transfer. CMV3/zeo HA-F-RhoB and CMV/zeo HA-GG-RhoB were a kind gift of Professor George Prendergast, Lancaster Avenue, Wynnewood, PA). The last four C-terminal amino acids of the F-RhoB were CLVS, while in GG-RhoB, 16 terminal residues of RhoB were replaced C by 13 C terminus of RhoA, with the last four residues CLVL.^3^ Adenoviral constructs for the farnesylated-RhoB (HA-F-RhoB; Ad-teto-RhoB-mut-F-GFP), geranylgeranylated-RhoB (HA-GG-RhoB; Ad-teto-RhoA/B-GG-GFP) as well as adenoviral control (Ad-GFP) were made using Ad5 ΔE1ΔE3 backbone vector (Welgen Worcester, MA USA). Briefly, the cDNA of RhoB-F and RhoB-GG was inserted into pENTCMV3-GFP vector pre-digested with Hind3/Xba1 respectively. The positive clones were screened with EcoR1 and sequenced. The pENT-RhoBmut-GG-GFP, pENT-RhoBmut-NG-GFP and pENT-RhoBmut-FF-GFP were treated with LR Clonase II enzyme (Invitrogen) and ligated to a pAd-REP plasmid that contains the remaining adenovirus genome. The recombination products were transformed into E. coli. After incubation overnight, the positive clones were selected, and cosmid DNA were purified. The purified cosmid DNA (2 mg) was digested with Pac1 and then transfected into 293 cells with Lipofectamine 2000 according to manufacturer’s instructions. The adenovirus plaques were seen 7 days after transfection. All adenoviruses were amplified in HEK293 cells and subsequently purified on 2 sequential caesium chloride gradients and then passed through PD10 columns (GE Healthcare) to reduce the salt concentration. The titre of Ad-HA-RhoB-F and Ad-HA-RhoB-GG was 2 x 10^11^ pfu (plaque forming units)/ml and 4x x 10^11^ pfu/ml respectively. Mutant protein expression was confirmed by immunofluorescence and Western blotting

Adenoviral infection was carried out at MOI (multiplicity of infection) 1:100.

BrdU incorporation measurement. Proliferation HPAECs and HPASMCs grown in 96-well plates was evaluated using bromodeoxyuridine (BrdU) assay (Millipore), according to the manufacturer’s recommendations.

HPAECs grown in full EGM-2 medium were infected with adenoviruses to overexpress AdGFP, AdF-RhoB or AdGG-RhoB. BrDU assay was carried out 18 hours post-infection. HPASMCs were infected with adenoviruses and pre-starved for 6 hours in culture medium containing 0.1% FCS before the addition of PDGF-BB (eBioscience, 20 µg/L) and BrdU. Following an overnight incubation, BrdU proliferation assay (Milipore) was carried out. As the overexpression of RhoB prenylation mutants induced actomyosin contractility and cell rounding, the 450nm absorbance value was normalised to the number of cells in untreated controls.

Fluorescent staining of BrdU-positive nuclei was carried out on cells fixed in 4% formaldehyde in PBS, according to the BrdU Labeling and Detection Protocol recommended by ThermoFisher Scientific (<https://www.thermofisher.com/uk/en/home/references/protocols/cell-and-tissue-analysis/protocols/brdu-labeling-and-detection-protocol.html>). The cells were incubated with primary mouse monoclonal Pierce anti-BrdU antibody (Life Technologies, MA1-82088, 10µg/mL), washed and incubated with a secondary FITC-labelled goat anti-mouse antibody (Jackson ImmunoResearch Laboratories, 115-095-062; 10µg/mL) together with 1mg/L of TRITC-phalloidin (Sigma) to stain actin. Coverslips were washed 3x in PBS and mounted in Vectashield mountant containing nuclear stain DAPI.

Label Free Proteomics. Cell lysates from HPAECs overexpressing RhoB mutants were prepared in 9M Urea. Protein content was estimated using the bicinchoninic acid method (Thermo Fischer Scientific, Cramlington, UK) with bovine albumin as standard. SDS-PAGE was performed using 10% NuPAGE Novex bis-tris gels and reagents (Invitrogen Ltd., Paisley, UK). Samples (n=5 per group) were reduced by addition of 50 mmol/L L-dithiothreitol prior to heating at 100°C for 2 min and then carbamidomethylated by addition of 200 mmol/L iodoacetamide in the dark for 30 min. Gels were stained with InstantBlue® and each sample-containing lane cut into a series of regions (R1-R14) based on the position of molecular weight (MW) markers (Spectra™, Multicolar Broad Range, Life Technologies) and the distribution of proteins observed in the gel. Each gel piece was then digested with trypsin, peptides extracted, and dried as described previously.^4^ Lyophilised samples were reconstituted in 30 µL of 0.1% TFA and 10 µL of the solution was injected onto a C18 trap-column (ProteCol, 0.3 x 10 mm, 300Å; SGE Analytical Science Pty Ltd. Victoria, Australia) followed by reverse phase separation on a C18 column (PicoFrit, 75µm ID × 10 cm ProteoPrep column, New Objective Inc., Massachussetts, USA) using Agilent 1200 LC series (Agilent Technologies UK Ltd., Berkshire, UK) as previously described.^4^ MS data was assessed on the basis of ion intensity of peptide ions with coincident LC retention time and m/z values as previously described.^4^ Protein identification was based on the presence of ≥2 unique peptides. Protein abundance values were calculated on the basis of the ion intensities of the component peptides. These data were displayed as an intensity map by Progenesis LC-MS software (Nonlinear Dynamics, Newcastle upon Tyne, UK). As the criterion of protein identification applied was based on detection ≥2 unique peptides, values for every peptide in all samples resulted; i.e. no data was excluded. Consequently, statistical comparisons based on parametric statistics were applied for each protein in all samples. Differentially expressed proteins were determined by identifying proteins with at least 2 unique peptides, ≥1.5 fold difference and P<0.05 (Student’s t-test).

Functional associations of the identified proteins were analysed with STRING (Search Tool for the Retrieval of Interacting Genes/Proteins version 10.0) database and Ingenuity Pathway Analysis version 01-07.

**Supplemental Data**

Figure S1


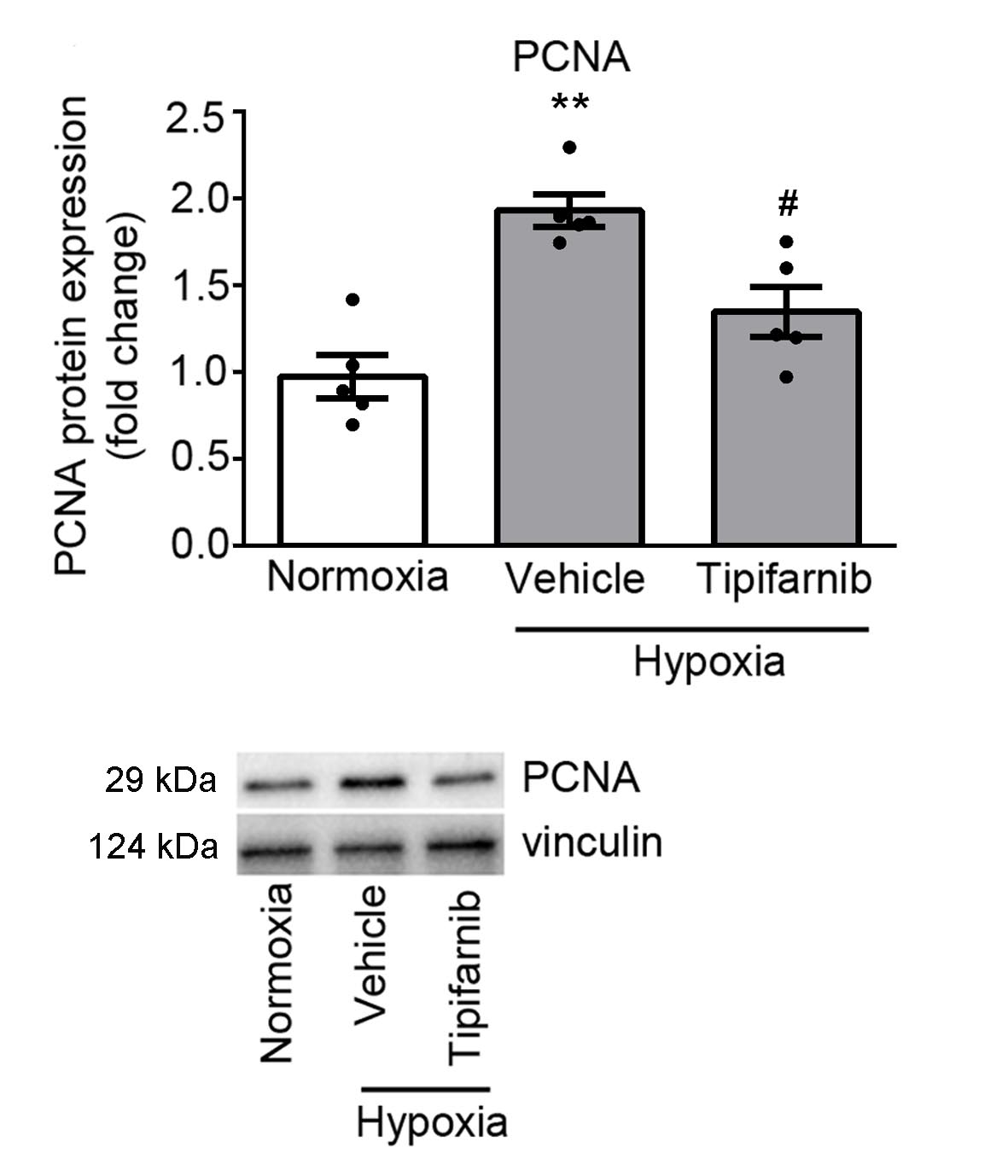


**Figure S1. Tipifarnib reduces PCNA expression in lungs of chronically hypoxic mice.**

Graph and representative western blot show PCNA protein expression in the lungs of normoxic and chronically hypoxic mice treated with vehicle or tipifarnib (100 mg/kg). Data represent mean±SEM of n=5. **p<0.01 vs normoxic control; #p<0.05 vs hypoxic control. 1-way ANOVA with Tukey post-test.

Figure S2


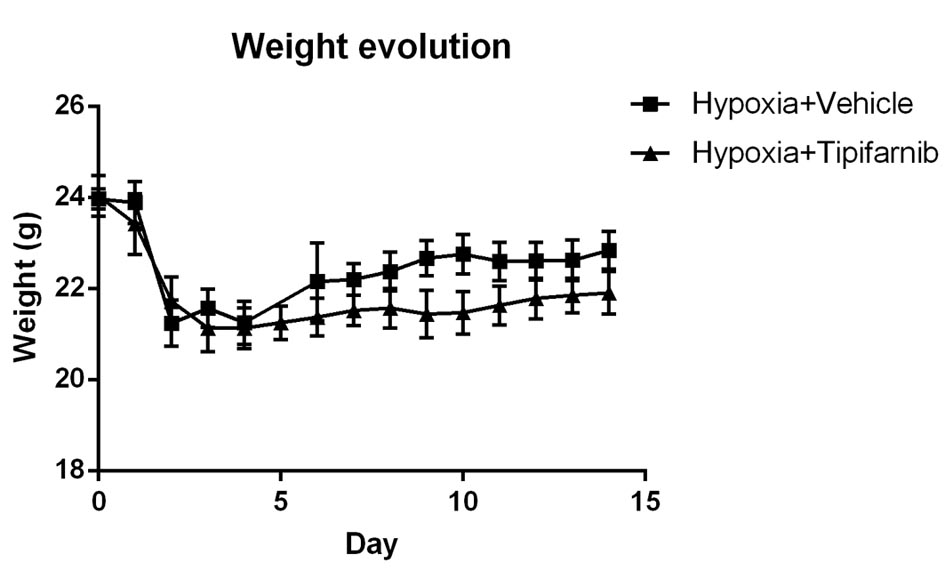


**Figure S2. Tipifarnib does not significantly affect mouse weight.** Mice were exposed to 2-week hypoxia and were given oral tipifarnib 100mg/kg twice daily (treatment group) or vehicle (hypoxic control group); n=8.

Figure S3


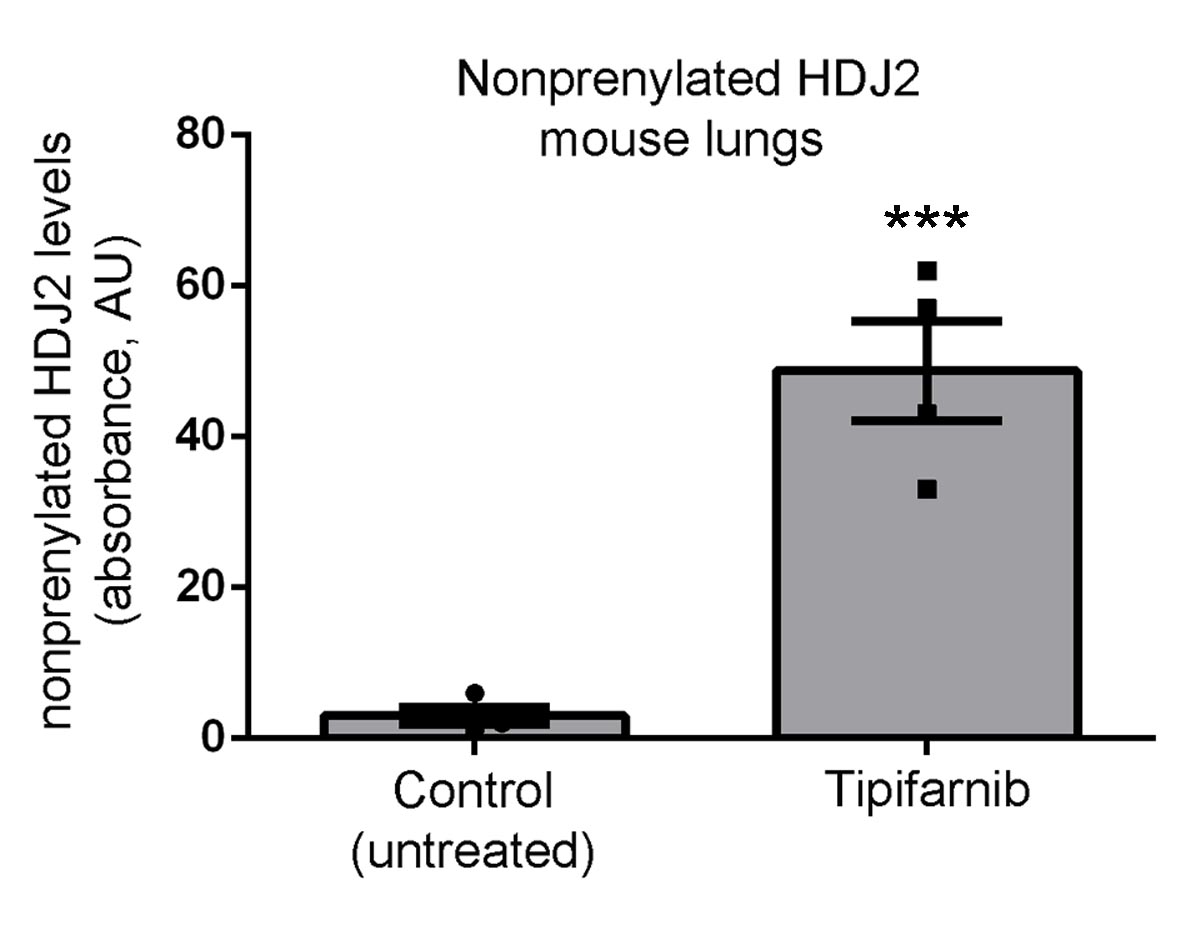


**Figure S3. Tipifarnib increases the levels of unprenylated HDJ2 in mouse lungs.** Mice were treated twice daily by oral gavage with either vehicle (2% carboxymethylcellulose in PBS) or tipifarnib (100 mg/kg/body weight).Treatments started one day prior hypoxia exposure. The effectiveness of tipifarnib in inhibition of protein farnesylation was confirmed by accumulation of nonprenylated form of HDJ2 (upper band of HDJ2 protein) in mouse lung lysates analysed by western blotting. Representative example of western blot is shown in Figure 2D; ***p<0.001, comparison with controls, unpaired t-test; n=4.

Figure S4


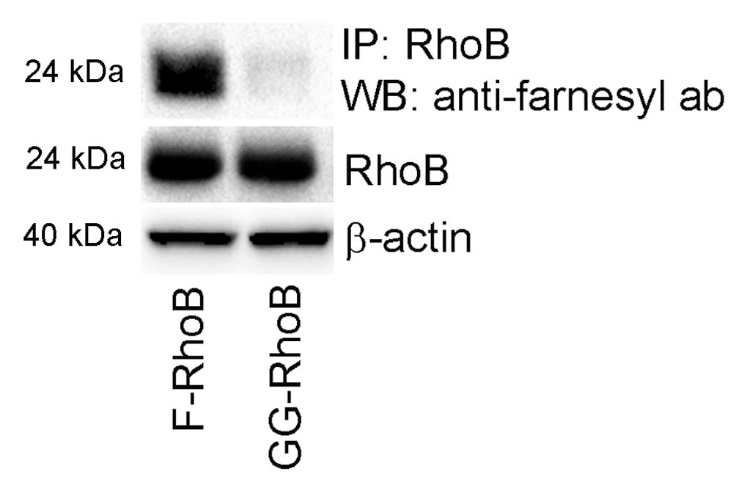


**Figure S4. Detection of F-RhoB and GG-RhoB with rabbit anti-farnesyl-proteins antibody (Milipore, AB4073).** HPAECs overexpressing F-RhoB or GG-RhoB were lysed and RhoB was immunoprecipitated with rabbit anti-RhoB antibody. Total cell lysates and sepharose beads with immunoprecipitated RhoB were resolved by electrophoresis followed by western blotting. The blots were then probed with a rabbit anti-farnesylated proteins antibody, anti-RhoB and anti-β-actin antibodies.

Figure S5.


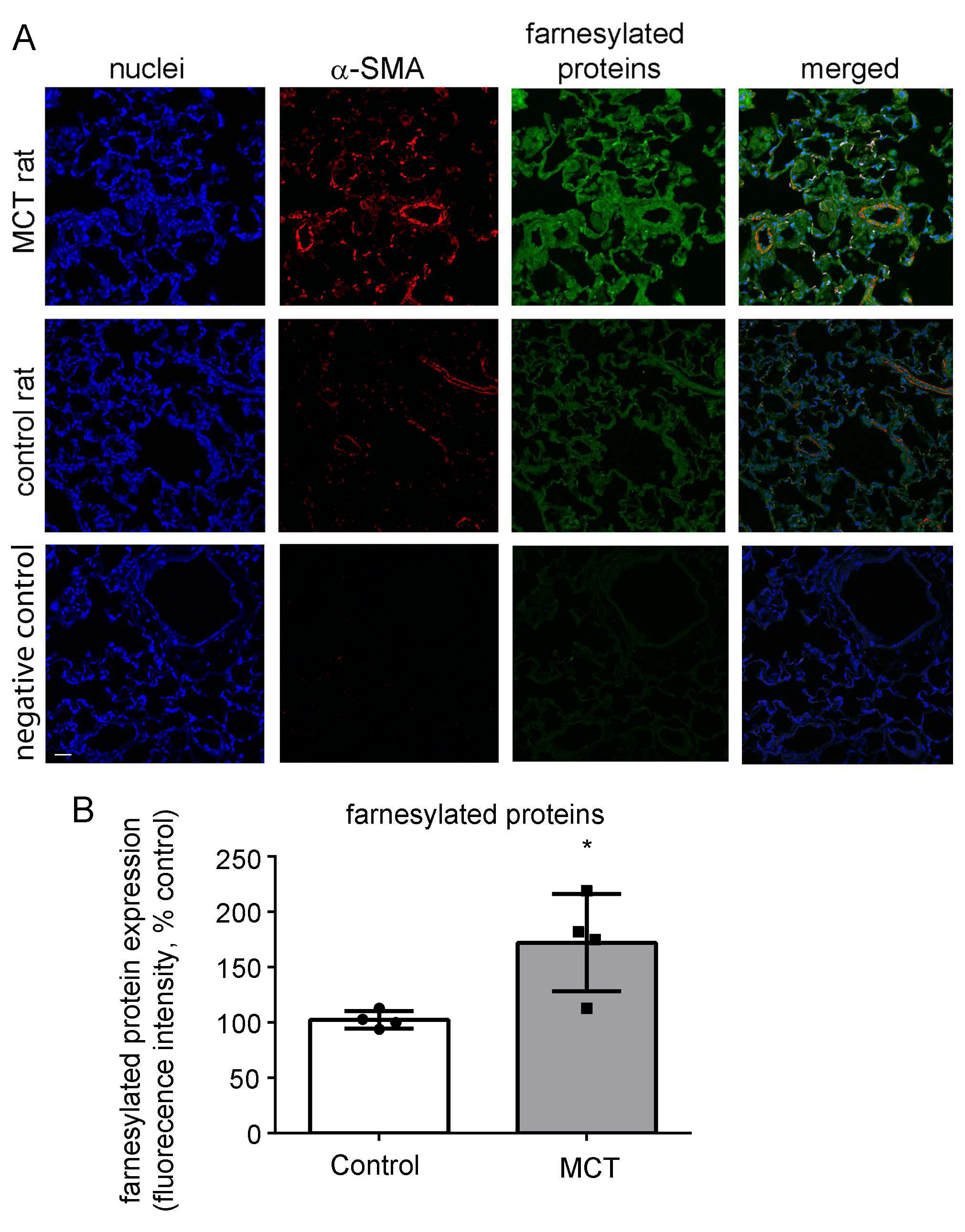


**Figure S5**. **Increased protein farnesylation in MCT rat lung.** (A) Representative immunofluorescent images showing cell nuclei (blue), farnesylated proteins (green) and α-smooth muscle actin (red) in MCT rat lung and control, healthy lung, as indicated. In the negative control, the tissues were incubated with the secondary antibodies only. All images were taken with the same settings of laser power and gain. Bar=10µm. (B) Change in the levels of farnesylated proteins in MCT lung. Fluorescence intensity was measured in 3 random fields of 4 healthy control lungs and 4 MCT lungs and results are expressed as % of healthy control values ±SEM. *p<0.05, unpaired t-test.

Figure S6


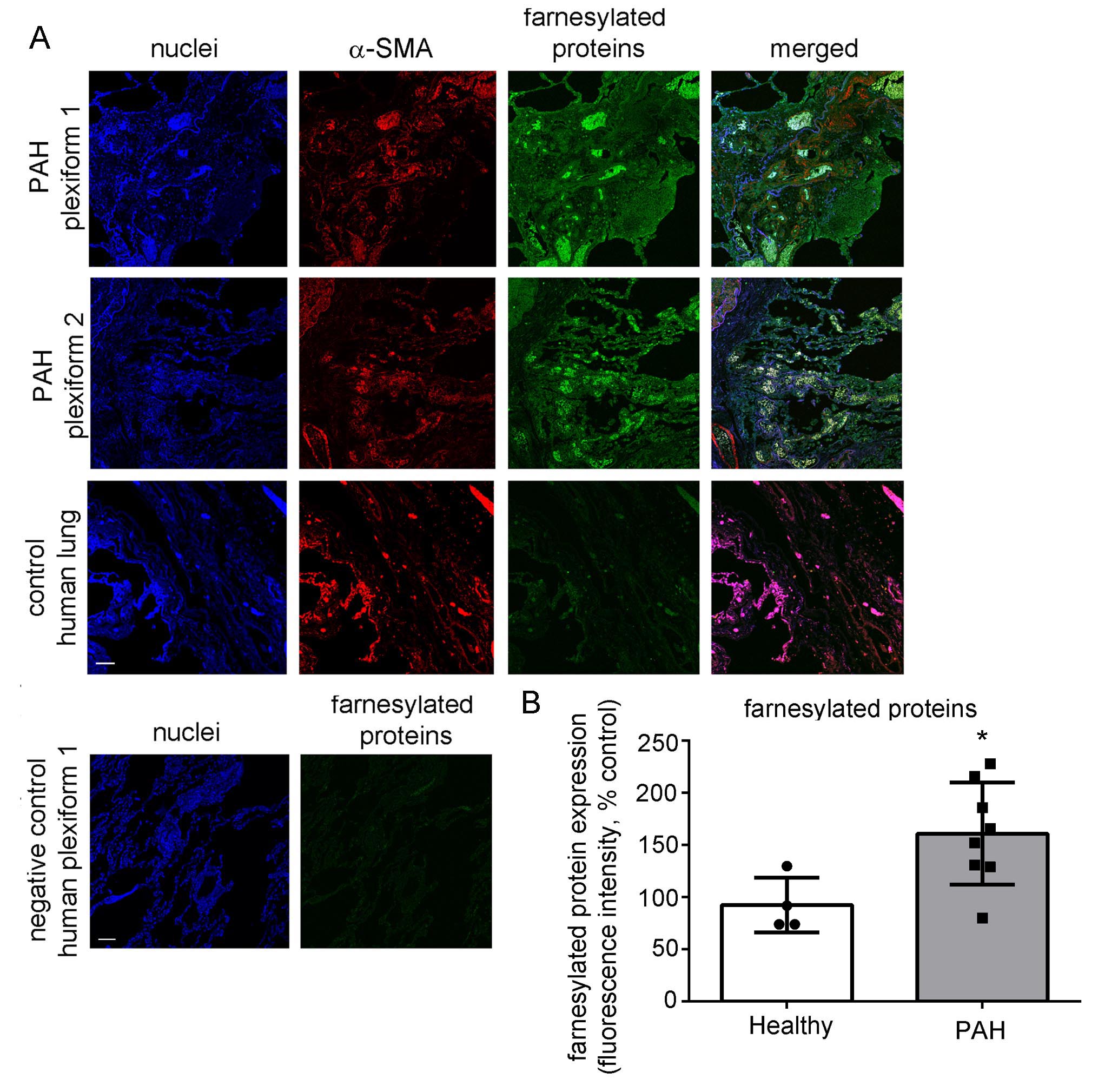


**Figure S6.** **Elevated protein farnesylation in PAH lungs.** (A) Representative immunofluorescent images showing cell nuclei (blue), farnesylated proteins (green) and α-smooth muscle actin (red) in PAH lung and control, healthy lung tissue, as indicated. In the negative control, the tissues were incubated with the secondary antibody only. All images were taken with the same settings of laser power and gain. Bar=10µm. (B) Increased levels of farnesylated proteins in PAH lung. Fluorescence intensity was measured in 3 random fields of 4 healthy control lungs and 8 PAH lung tissues and results are expressed as % of healthy control values ±SEM. *p<0.05, unpaired t-test.

Figure S7


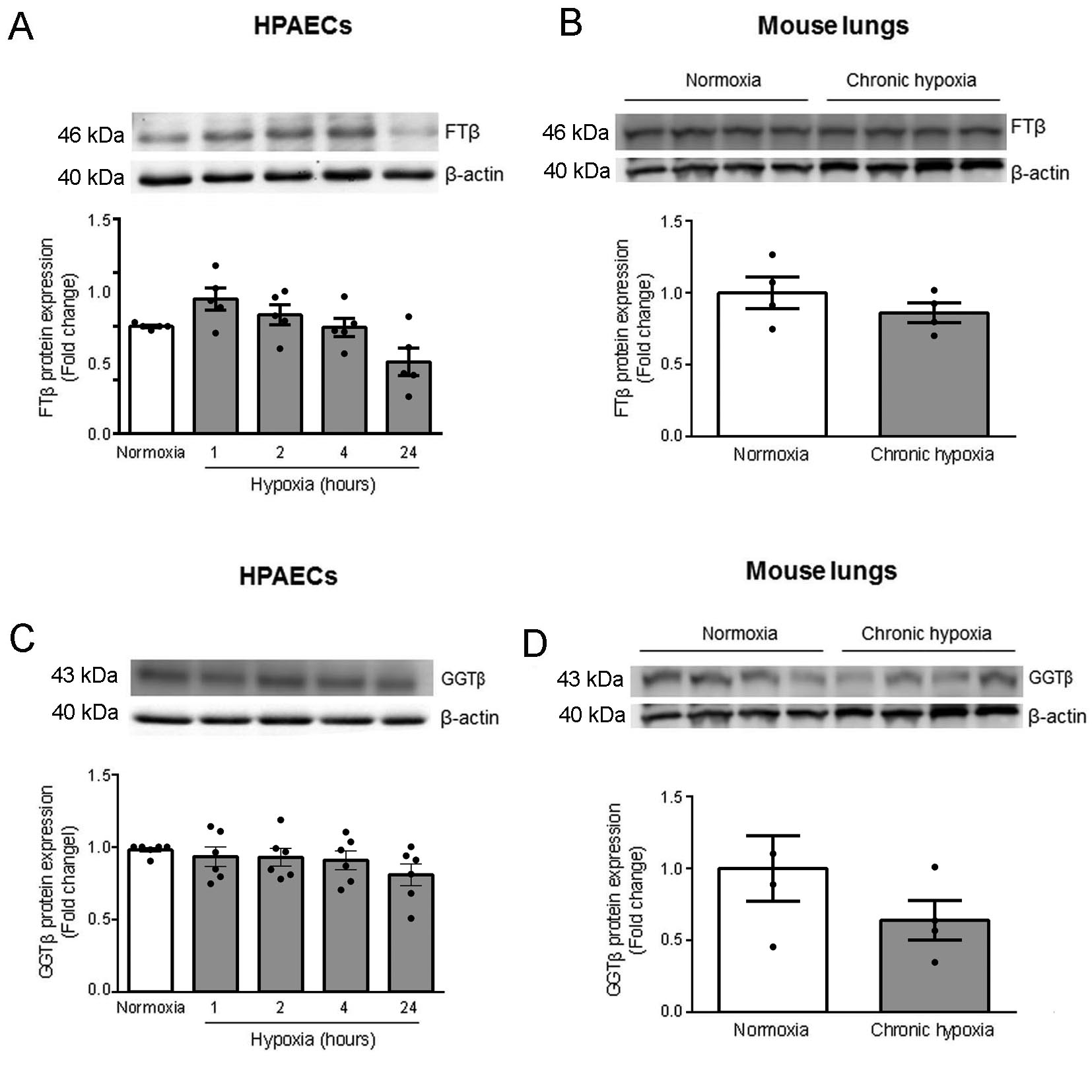


**Figure S7. Hypoxia does not affect farnesyltransferase and geranylgeranyltransferase protein expression *in vitro* or *in vivo*.** Protein expression of farnesyltransferase subunit beta (FTβ) in (A) HPAECs exposed to hypoxia for 1-24 hours and (B) in lungs of mice exposed to chronic hypoxia for 2 weeks. (C) and (D) show protein expression of geranylgeranyltransferase subunit beta (GGTβ) in HPAECs and hypoxic lung tissues, as indicated. Representative western blots are shown above the graphs. Densitometric analysis of western blots; β-actin expression was used as sample loading control. Results are expressed as mean fold- change over control ±SEM. In (A, C) n=5-6 and in (B, D) n=4.

Figure S8

**
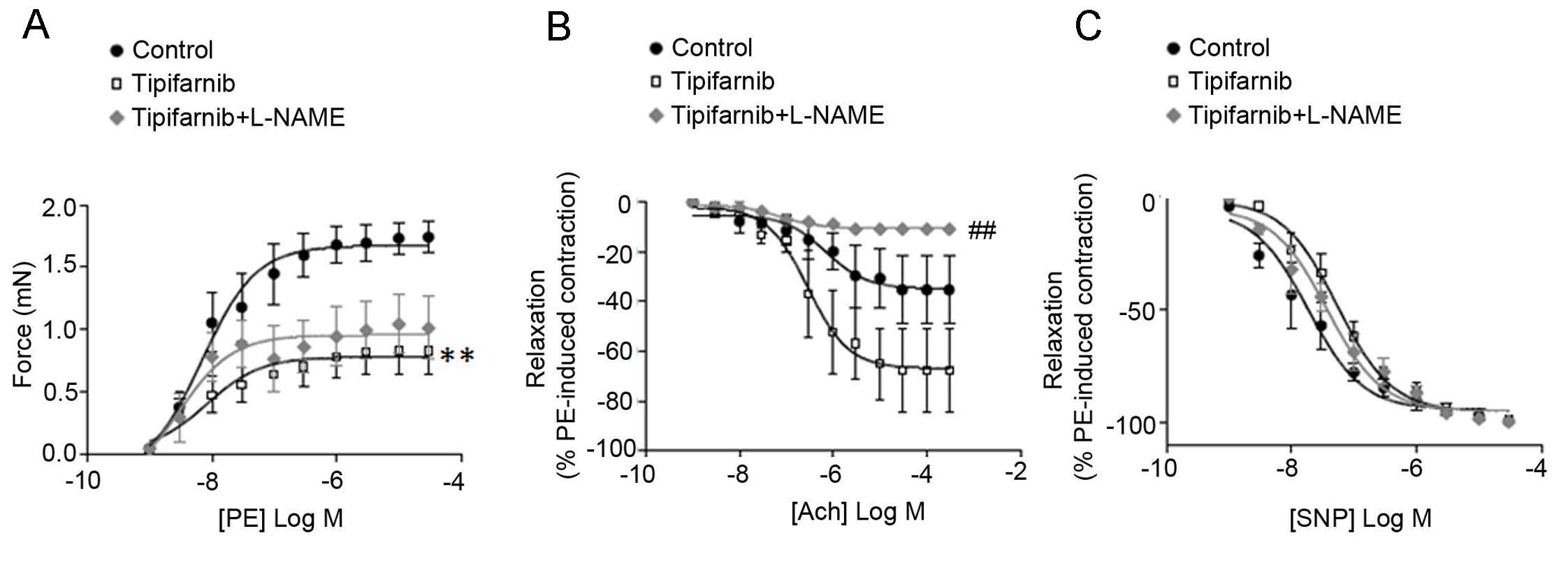
**

**Figure S8**. **L-NAME prevents tipifarnib-induced increase in vasorelaxation.** (A) Contractile response to phenylephrine, (B) relaxant response to acetylcholine and (C) relaxant response to SNP, were assessed in mouse intrapulmonary arteries pretreated with 0.1 μmol/L tipifarnib for 2 hours and 100 μmol/L L-NAME for 30 minutes prior to the start of the experiment. Data represent mean ± SEM of n=4, 0% relaxation corresponding to the level of precontraction induced by 3x10^-8^ M phenylephrine. Statistical significance was determined using a (A-C) two-way ANOVA with repeated measures and a Bonferroni post-hoc test. **p<0.01 vs control; ##p<0.01 vs tipifarnib.

Figure S9.


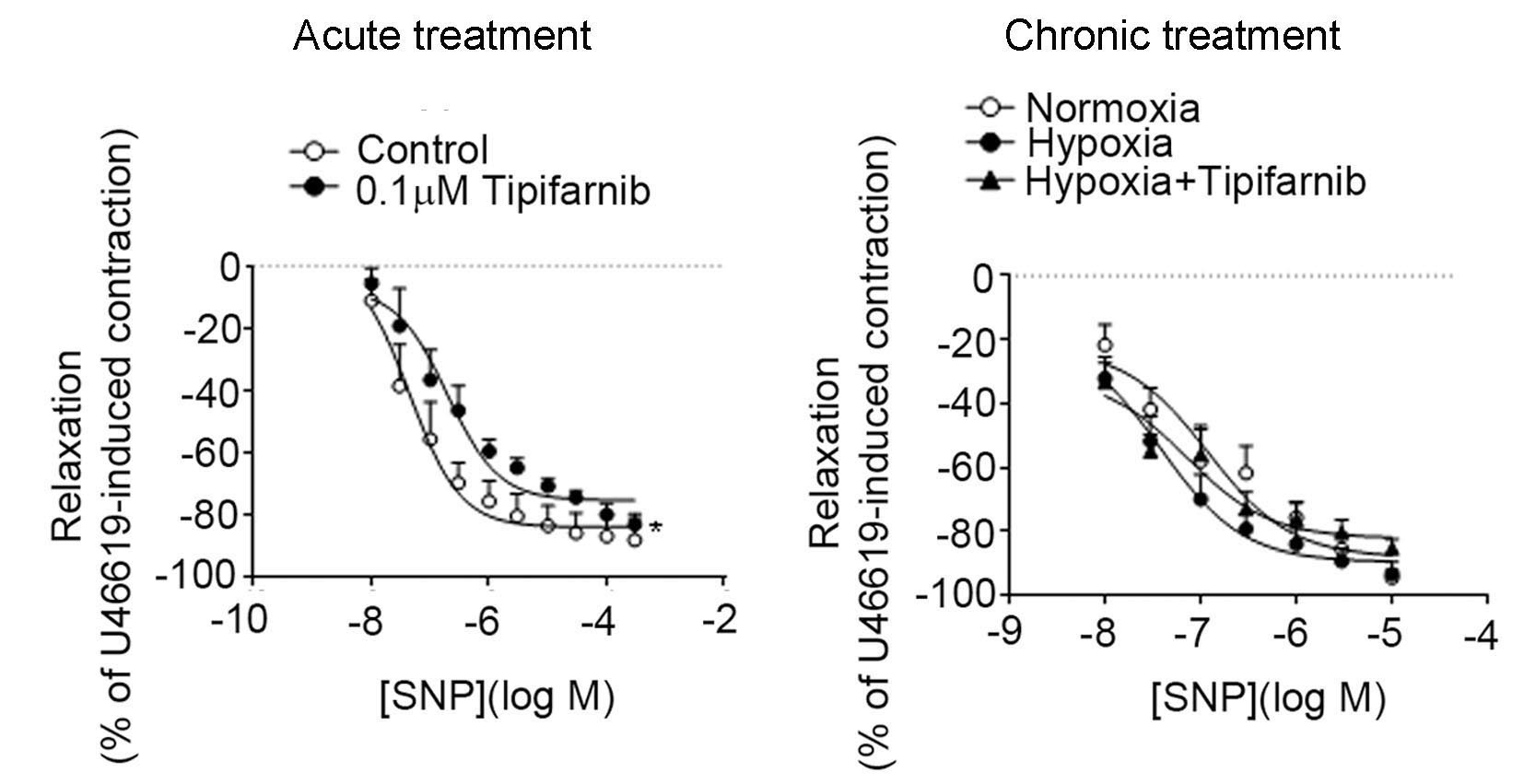


**Figure S9.** **The effect of tipifarnib on relaxant response to SNP.** Relaxant responses of intrapulmonary arteries (A) pretreated for 2 hours with 0.1 μmol/L tipifarnib (Acute treatment) or (B) isolated from normoxic, hypoxic and hypoxic tipifarnib-treated mice (Chronic treatment). Data represent mean ± SEM of n=4 for acute treatment and n=8-12 for chronic treatment, 0% relaxation corresponding to the level of precontraction induced by 3x10^-6^ mol/L U44162. *p<0.05, comparison with control. Statistical significance was determined using a two-way ANOVA with repeated measures and a Bonferroni post-hoc test.

Figure S10.


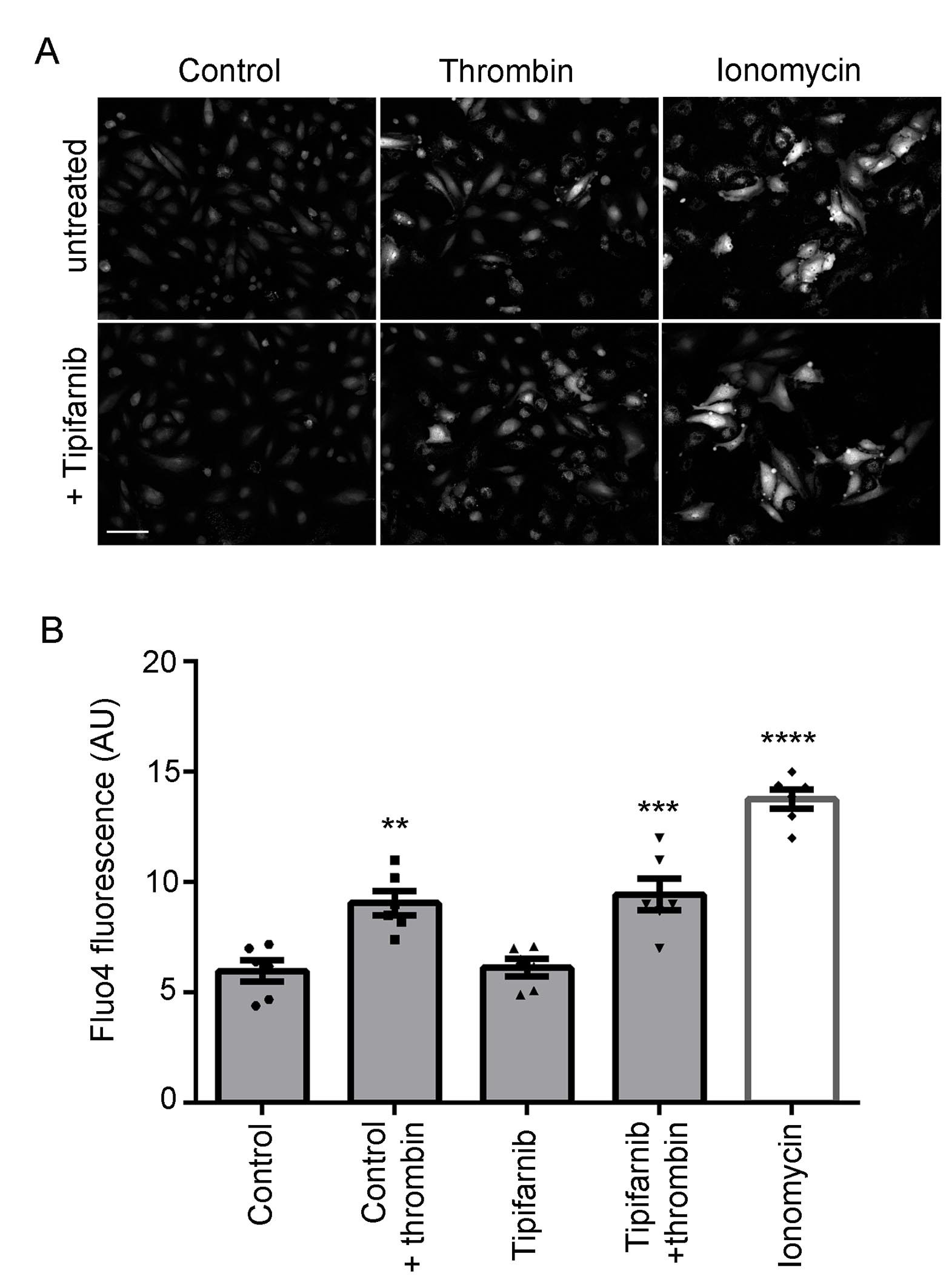


**Figure S10. Tipifarnib does not change intracellular calcium levels in HPAECs**. HPAECs were left untreated or were treated with 0.1µmol/L tipifarnib for 24 hours. Calcium flux was induced by thrombin (1U/mL) or ionomycin (1µmol/L) for 3 minutes. Intracellular calcium levels were studied with Fluo4 NW Calcium Assay kit (Invitrogen, F36206), according to the manufacturer’s protocol. Images of cells were taken under the confocal laser scanning fluorescence microscope (Leica TCS SP5) and the intensity of Fluo4 fluorescence (excitation 494 nm and emission 516 nm) was measured with Image J. **p<0.01, ***p<0.0001, comparison with untreated controls, 1-way ANOVA, Dunnett’s post-test; n=5.

Figure S11.


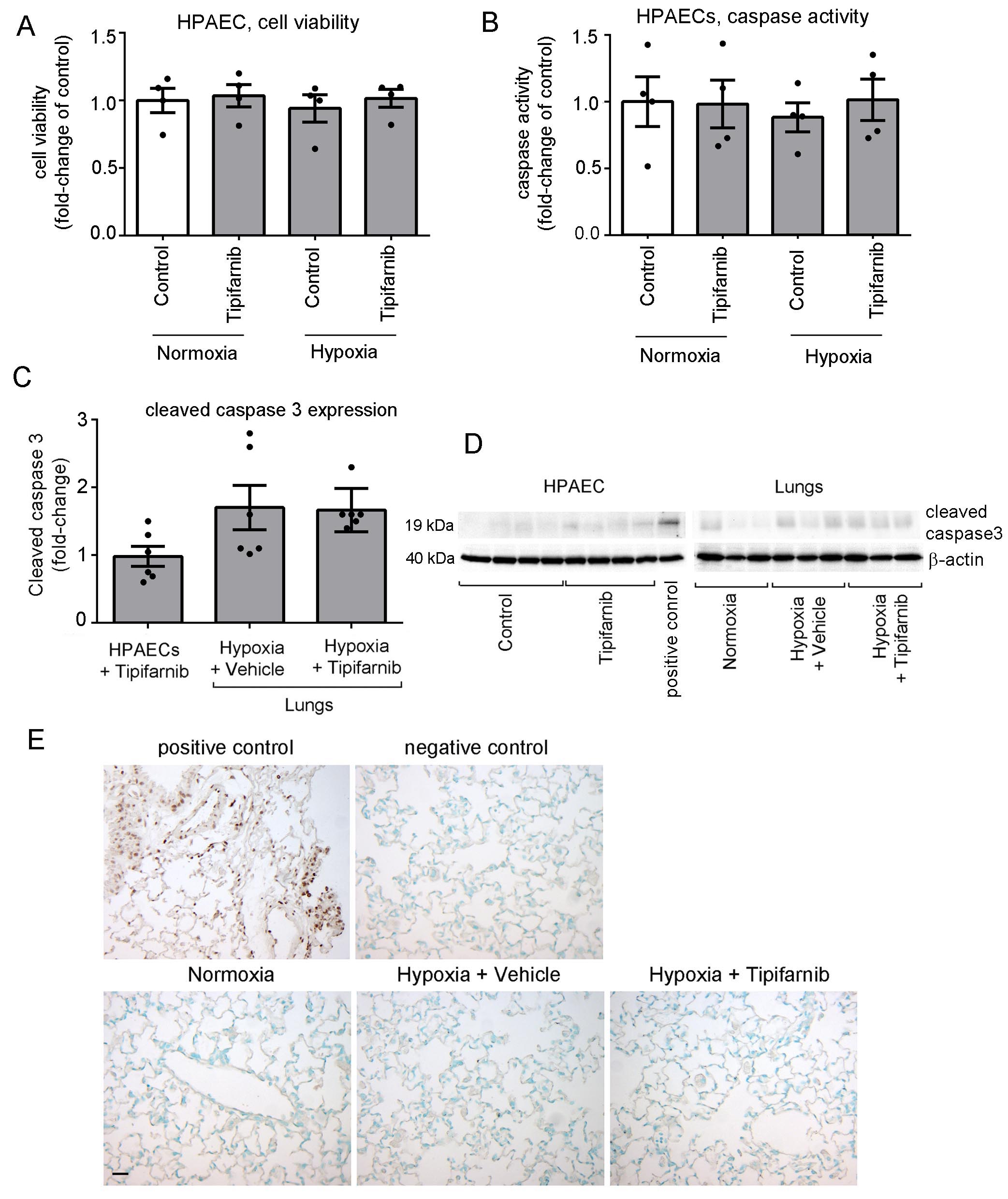


**Figure S11. Tipifarnib does not affect cell viability or apoptosis.** (A) Cell viability and (B) caspase activation were measured in multiplexed assay ApoTox-Glo™ assay in HPAECs stimulated with tipifarnib (0.1 µmol/L) and exposed to hypoxia for 24 hours. Results are expressed as mean fold- change over control±SEM, n=4. Graph in (C) and corresponding representative western blots in (D) show changes in protein levels of cleaved caspase 3 in HPAECs treated with tipifarnib (0.1 µmol/L), lungs of hypoxic mice (Hypoxia+Vehicle) and lungs of hypoxic mice treated with tipifarnib (Hypoxia+Tipifarnib). In (C) cleaved caspase expression is shown as fold-change over control untreated HPAECs or control normoxic lungs, as appropriate ± SEM; n=6. Broken line indicates control level. Cell lysate of menadione-treated HPAECs (50µmol/L, 6hours) was used as a positive control. (E) Immunohistochemical analysis of apoptotic DNA fragmentation in mouse lungs with TACS 2 TdT DAB *in situ* Apoptosis Detection kit (Trevigen). Apoptotic nuclei are brown with Methyl Green counterstain. In positive control TACS-Nuclease was added to the labelling mix to generate DNA breaks. Negative control did not have dTdT enzyme in the labelling mix. Bar=50µm. Images are representative of n=4.

Figure S12.


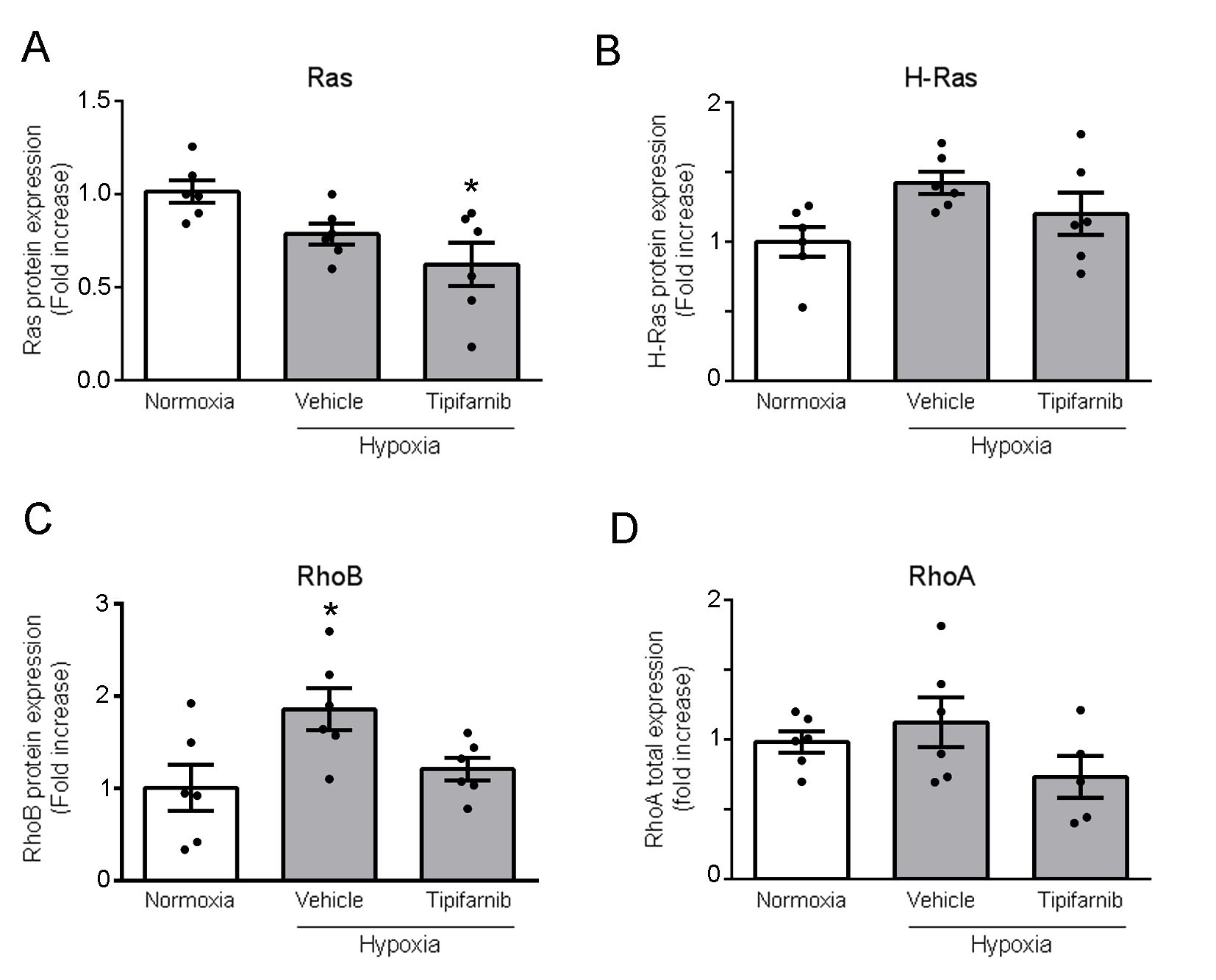


**Figure S12.** **Tipifarnib reduces protein expression of Ras and RhoB in lungs of hypoxic mice.** (A) Ras, (B) H-Ras, (C) RhoB and (D) RhoA protein expression levels were studied in the lungs of normoxic mice or hypoxic mice treated with vehicle or tipifarnib. Data represent mean±SEM of n=5-6. *p<0.05, vs normoxic control.1-way ANOVA with Dunnett’s post-test.

Figure S13.


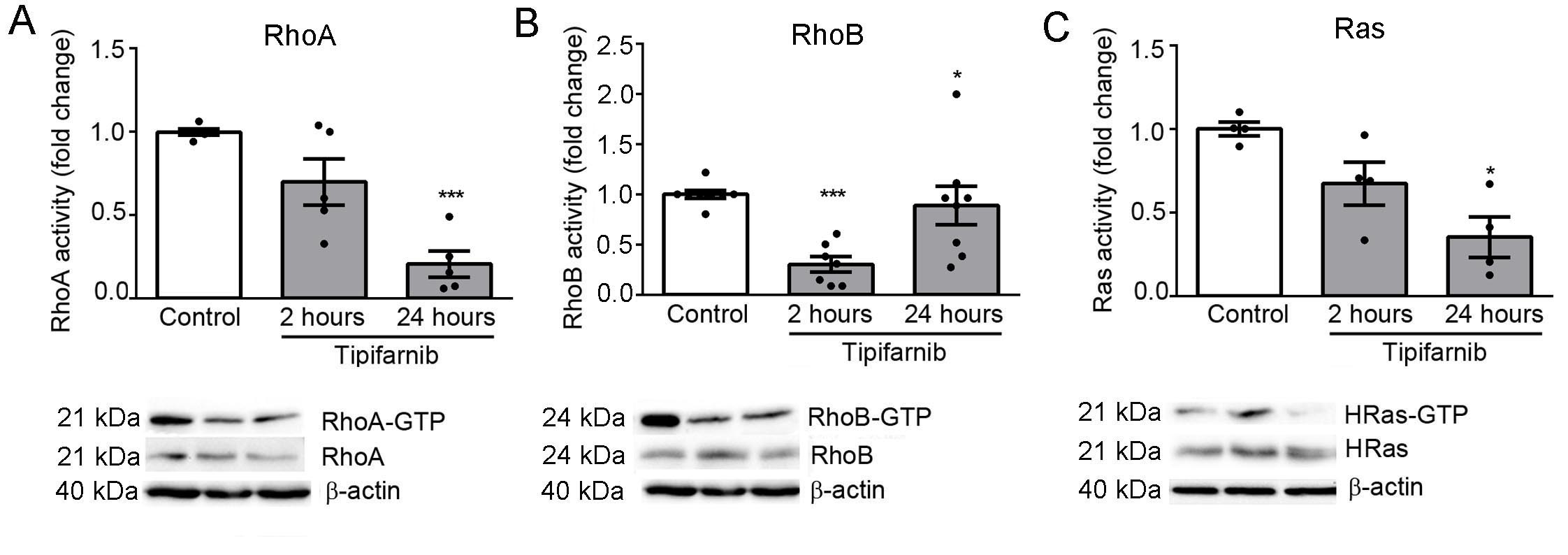


**Figure S13.** **Farnesyltransferase inhibition reduces the activities of RhoA, RhoB and Ras in HPAECs.** Activities of (A) RhoA, (B) RhoB and (C) Ras were assessed in HPAECs treated with tipifarnib for 2 hours or 24 hours, as indicated. β-actin was used as a normalization control. Results are expressed as fold-change of controls. Data represent mean±SEM of n=4-7. *p<0.05, **p<0.01 ***p<0.001 vs control. 1-way ANOVA with Tukey post-test.

Figure S14


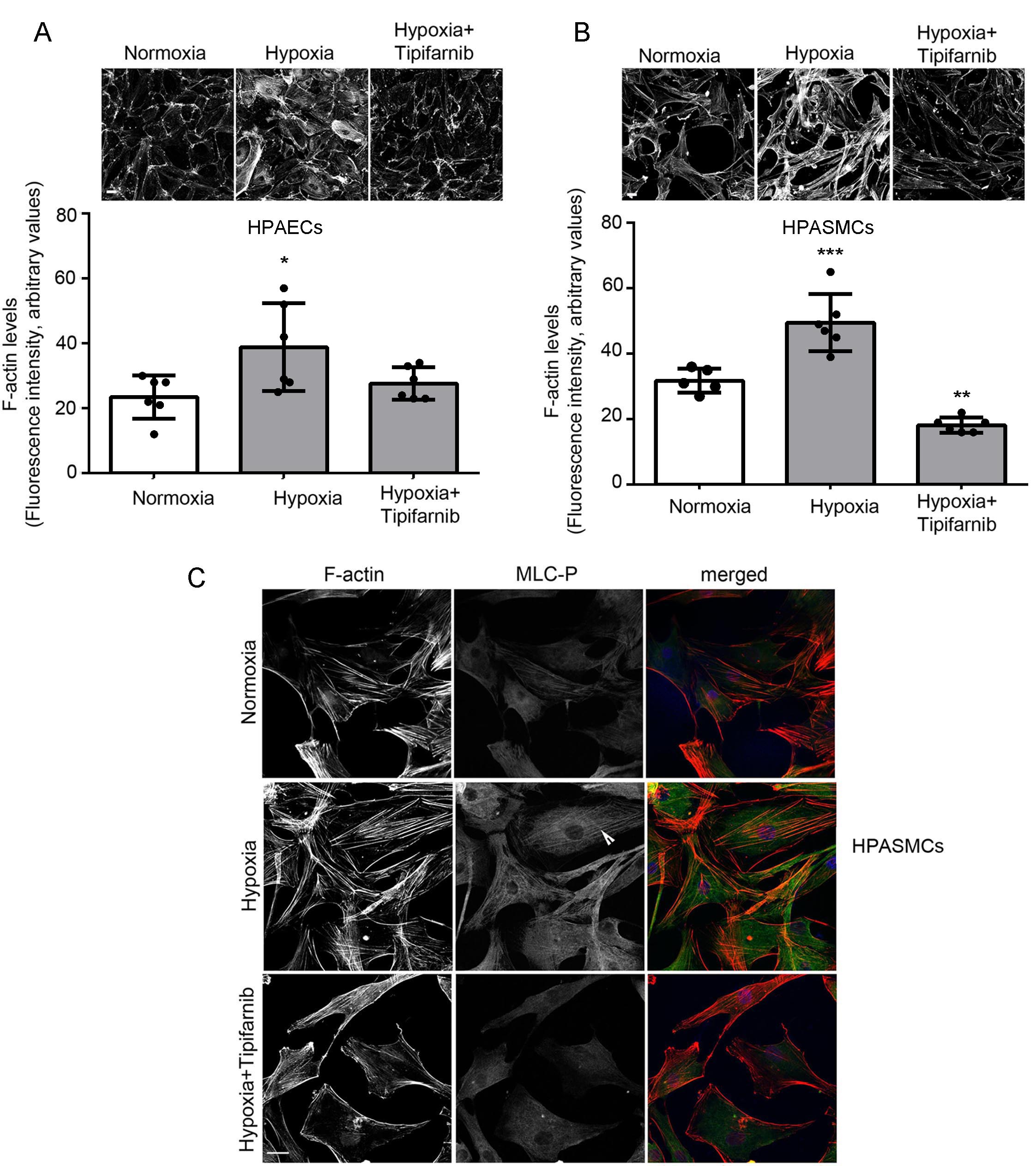


**Figure S14. Tipifanib prevents hypoxia-induced increase in F-actin levels in HPAECs and HPASMCs.** (A) HPAECs or (B) HPASMCs were left untreated or were treated with 0.1 µmol/L tipifarnib and exposed to hypoxia for 2 hours. F-actin was stained with TRITC-phalloidin and the intensity of fluorescence was measured in confocal images with Image J. Representative images of cells are shown above the graphs. Values are means±SEM of n=5-6. *p<0.05; **p<0.001, comparison with normoxic controls. 1-way ANOVA with Tukey post-test. (c) Tipifarnib inhibits the localization of phosphorylated MLC to stress fibres in hypoxic HPASMCs; confocal microscopy. The arrowhead points to p-Ser19 MLC co-localising with F-actin fibres in hypoxic HPASMCs. In merged images, F-actin is red, p-Ser19 MLC is green and nuclei are blue. Bar=10µm.

Figure S15.


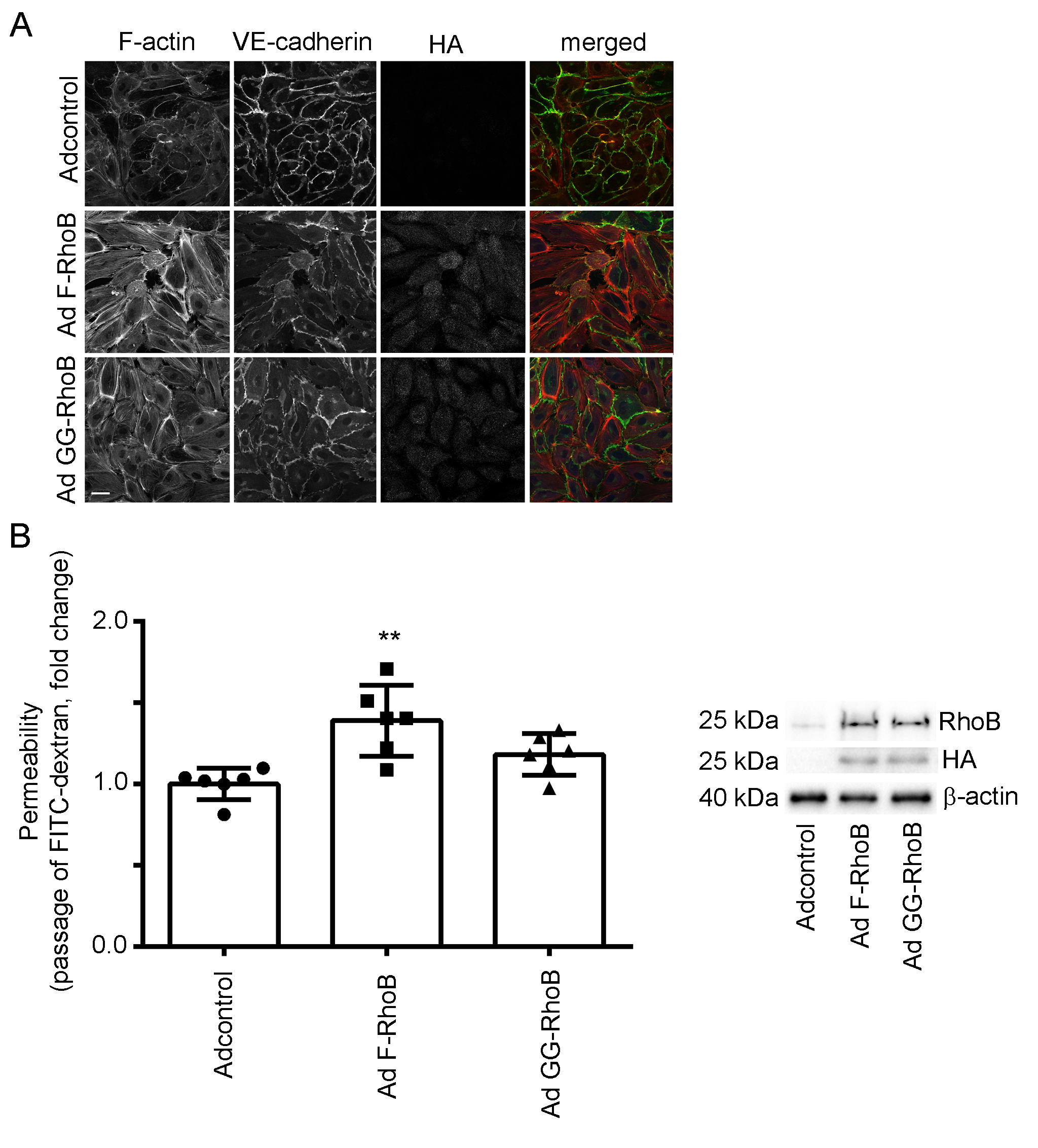


**Figure S15.** **Farnesylated RhoB increases endothelial permeability in HPAECs**. HPAECs were infected with AdGFP (Adcontrol), F-RhoB or GG-RhoB. 6 hours post-infection, endothelial permeability was measured as passage of fluorescent dextran across HPAEC monolayer; fold-change of controls. Values are means±SEM of n=6. **p<0.01; comparison with adenoviral controls. 1-way ANOVA with Tukey post-test.

Figure S16.


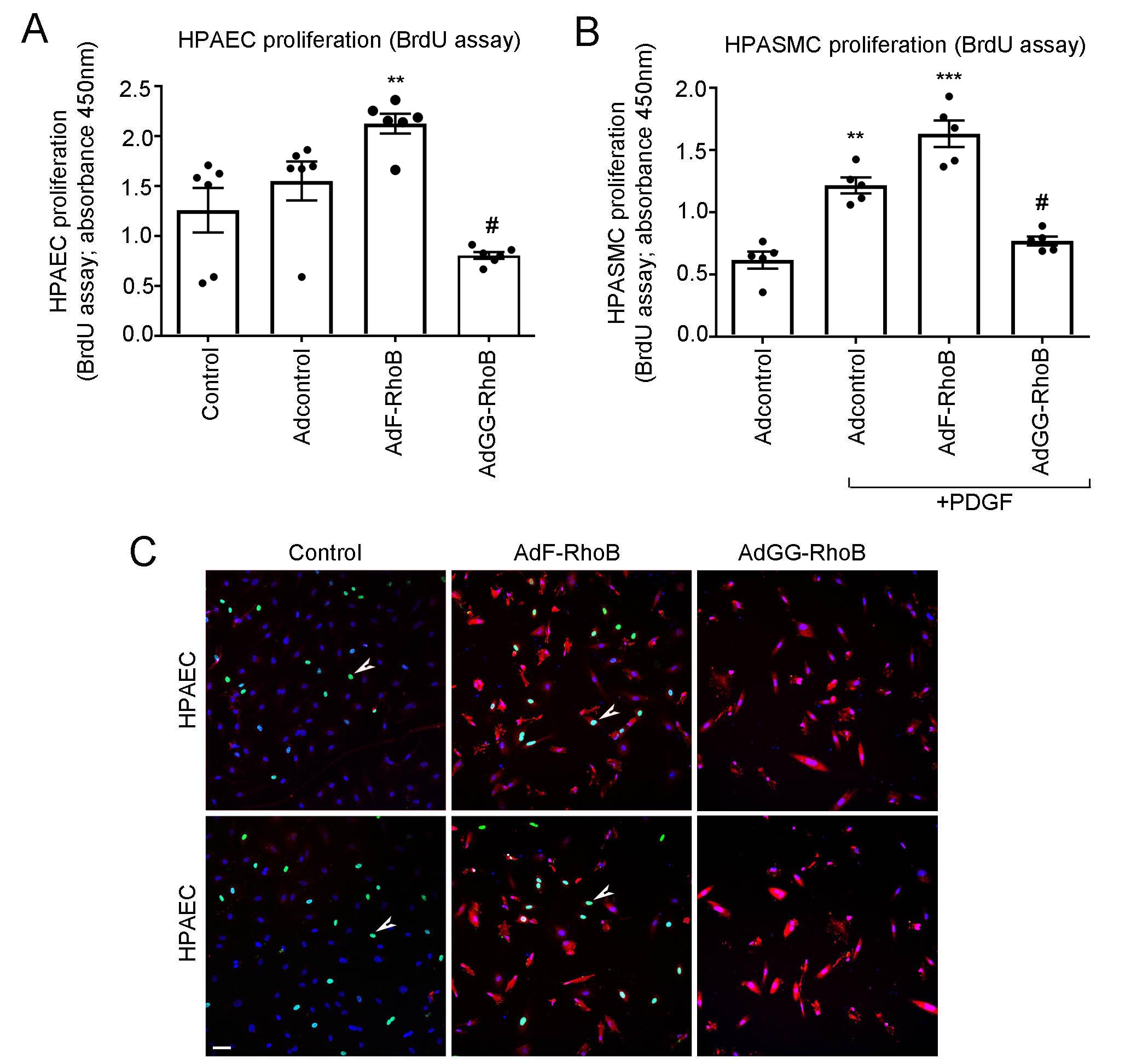


**Figure S16**. Farnesylated RhoB stimulates BrdU incorporation in HPAECs and HPASMCs. (A) BrDU incorporation in HPAECs overexpressing AdGFP (Adcontrol), AdF-RhoB or AdGG-RhoB for 18 hours; (B) BrDU incorporation in HPASMCs overexpressing AdGFP or RhoB mutants and treated with 20 ng/mL of PDGF-BB, as indicated. HPASMCs were pre-starved for 6 hours in culture medium containing 0.1% FCS, infected with adenoviruses and incubated with PDGF for 18 hours before BrdU proliferation assay (Milipore). The 450 nm absorbance value was normalised to the number of cells in untreated controls. Values are means±SEM of n=5-6. **P<0.01, comparison with untreated controls (HPAECs) or with adenoviral controls (HPASMCs); ***P<0.001, comparison with adenoviral controls; ^#^P<0.05, comparison with Adcontrols (HPAECs) or PDGF-treated Adcontrols (HPASMCs), as appropriate. 1-way ANOVA with Tukey post-test. (C) Fluorescent staining of BrdU incorporation in untreated HPAECs or HPAECs overexpressing AdF-RhoB or AdGG-RhoB, as indicated. All nuclei are labelled with DAPI (blue), BrdU-incorporating nuclei are green (arrowheads) and F-actin is red. Bar=30µm.

Figure S17.


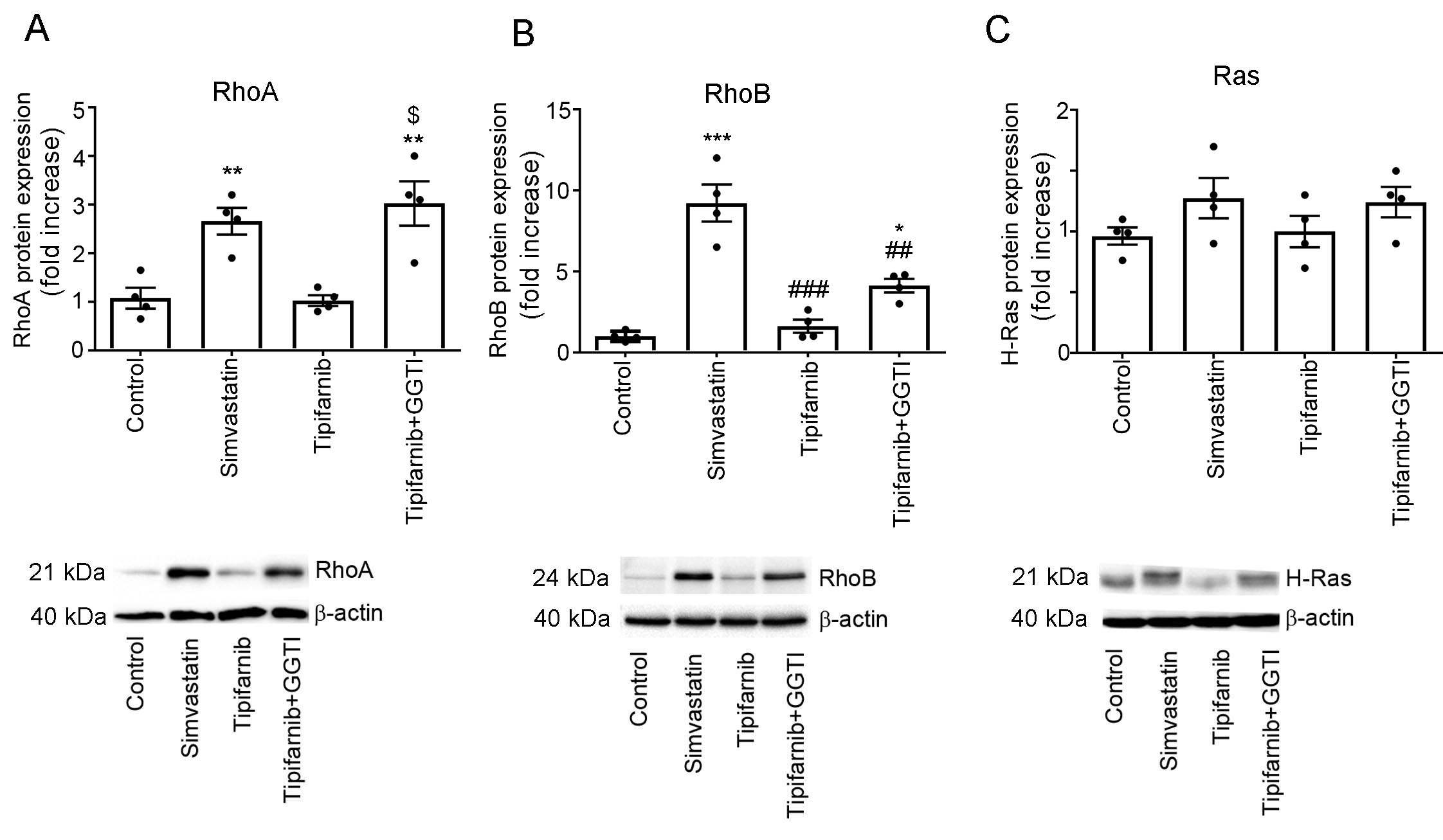


**Figure S17:** **Differential effect of simvastatin and tipifarnib on protein expression of Ras and Rho GTPases in HPAECs.** (A) RhoA, (B) RhoB and (C) H-Ras protein expression was studied in HPAECs incubated with simvastatin (10 µmol/L), tipifarnib (0.1 µmol/L), GGTi-995 (5µmol/L) or a combination of tipifarnib with GGTi-995 for 24 hours, as indicated. Results are expressed as fold change over control. Data represent mean ± SEM of n=4. ^*^p<0.05, ^**^p<0.01, ^***^p<0.001 vs control; ^##^p<0.01, ^###^p<0.001 vs simvastatin-treated cells; ^$^p<0.05 vs tipifarnib-treated cells. 1-way ANOVA with Tukey post-test. Corresponding representative western blots are shown below the graphs.

**Table S1:** Proteomic analysis of differentially expressed proteins in HPAECs overexpressing RhoB mutants: proteins upregulated in HPAECs overexpressing F-RhoB or GG-RhoB. (a) lists proteins increased in GG-RhoB-overexpressing HPAECs and (b) lists proteins increased in F-RhoB-overexpressing HPAECs. Only proteins with fold-increase >1.5 and p value <0.05, were listed. (+) or (-) indicate higher or lower protein levels in FF samples compared to GG. * Proteins involved in the regulation of cell metabolism identified with STRING

| **(a) Proteins increased in GG-RhoB-overexpressing HPAECs** | | | | | |
| --- | --- | --- | --- | --- | --- |
| **Protein** | **Ratio** | **p value** | **Adj.**  **p value** | **Protein accession number** | **Gene symbol** |
| Protein Jumonji* | -2.64 | 1.58E-02 | 0.16 | NP_004964 | JARID2 |
| Protein misato homolog 1 | -2.43 | 4.19E-02 | 0.27 | NP_060586 | MSTO1 |
| 182 kDa tankyrase-1-binding protein | -2.27 | 1.76E-03 | 0.09 | NP_203754 | TAB182 |
| Protein disulfide-isomerase A6* | -2.17 | 2.75E-03 | 0.07 | NP_005733 | PDIA6 |
| Disks large homolog 5* | -2.09 | 9.97E-03 | 0.14 | NP_004738 | DLG5 |
| Coiled-coil domain-containing protein 57 | -1.92 | 3.04E-02 | 0.25 | NP_932348 | CCDC57 |
| Kinesin-like protein KIF14* | -1.84 | 3.17E-02 | 0.25 | NP_055690 | KIF14 |
| Proteasome subunit beta type-3* | -1.80 | 4.59E-02 | 0.28 | NP_002786 | PSMB3 |
| Chromodomain-helicase-DNA-binding protein 7* | -1.68 | 3.68E-02 | 0.26 | NP_060250 | CHD7 |
| Beta-enolase | -1.67 | 3.37E-02 | 0.25 | NP_001967 | ENO3 |
| 26S protease regulatory subunit 7* | -1.65 | 4.99E-02 | 0.29 | NP_002794 | PSMC2 |
| Helicase SRCAP* | -1.64 | 3.81E-02 | 0.26 | NP_006653 | SRCAP |
| Spectrin alpha chain | -1.62 | 2.72E-02 | 0.23 | NP_003117 | SPTA1 |
| Hsc70-interacting protein* | -1.54 | 4.09E-02 | 0.27 | NP_003923 | ST13 |
| Importin-4 | -1.54 | 5.19E-02 | 0.29 | NP_078934 | IPO4 |
| Meiotic nuclear division protein 1 homolog | -1.54 | 4.94E-02 | 0.29 | NP_115493 | MND1 |
| **(b) Proteins increased in F-RhoB-overexpressing HPAECs** | | | | | |
| RANBP2-like and GRIP domain containing 6 | 8.60 | 3.95E-02 | 0.27 | NP_005045 | RGPD6 |
| Small G protein signaling modulator 2* | 5.80 | 1.38E-02 | 0.14 | NP_055668 | SGSM2 |
| CUB and sushi domain-containing protein 2 | 3.40 | 5.83E-04 | 0.03 | NP_443128 | CSMD2 |
| 3-hydroxyacyl-CoA dehydrogenase type-2 | 3.18 | 6.10E-04 | 0.03 | NP_004484 | HSD17B10 |
| Epidermal growth factor receptor substrate 15-like 1 | 2.65 | 2.73E-02 | 0.29 | NP_067058 | EPS15L1 |
| Caspase recruitment domain-containing protein 14* | 2.63 | 2.50E-02 | 0.22 | NP_077015 | CARD14 |
| synaptojanin-dependent RNA helicase DDX1 | 2.59 | 1.92E-02 | 0.19 | NP_004930 | DDX1 |
| Heat shock 70 kDa protein 1-like | 2.54 | 1.35E-03 | 0.04 | NP_005518 | HSPA1L |
| E3 ubiquitin-protein ligase RNF213 | 2.44 | 3.74E-02 | 0.32 | NP_001243000 | RNF213 |
| Plastin-3 | 2.42 | 1.63E-03 | 0.07 | NP_005023 | PLS3 |
| ATP-binding cassette sub-family F member 2 isoform* | 2.42 | 1.47E-04 | 0.04 | NP_005683.2 | ABCF2 |
| RNA polymerase II transcription factor SIII | 2.35 | 1.42E-03 | 0.04 | NP_057511 | TCEB3B |
| Serine/threonine-protein kinase DCLK2 | 2.32 | 3.06E-02 | 0.24 | NP_001035350 | DCLK2 |
| Gamma-tubulin complex component 6 | 2.31 | 4.80E-02 | 0.36 | NP_065194 | TUBGCP6 |
| Plastin-2* | 2.23 | 2.37E-02 | 0.28 | NP_002289 | LCP1 |
| Poly [ADP-ribose] polymerase 14 | 2.20 | 8.96E-03 | 0.12 | NP_060024 | PARP14 |
| Unconventional myosin-VI* | 2.15 | 1.48E-02 | 0.16 | NP_004990 | MYO6 |
| Transketolase | 2.14 | 7.59E-03 | 0.07 | NP_001055 | TKT |
| Transmembrane 9 superfamily member 3 | 2.14 | 4.75E-02 | 0.28 | NP_064508 | TM9SF3 |
| Protein CASC5* | 2.11 | 3.86E-02 | 0.26 | NP_733468 | CASC5 |
| EF-hand domain-containing family member B | 2.07 | 4.10E-02 | 0.37 | NP_653316 | EFHB |
| serine/threonine-protein phosphatase 2A 65 kDa | 2.04 | 8.65E-03 | 0.16 | NP_859050 | PPP2R1B |
| Dolichyl-diphosphooligosaccharide--protein glycosyltransferase* | 2.04 | 1.14E-02 | 0.11 | NP_002941 | RPN1 |
| Asparagine--tRNA ligase, cytoplasmic | 2.03 | 7.13E-03 | 0.15 | NP_004530 | NARS |
| Sperm flagellar protein 2* | 2.01 | 3.55E-02 | 0.32 | NP_079143 | SPEF2 |
| Plectin* | 2.00 | 1.32E-02 | 0.15 | NP_958781 | PLEC |
| Cytoskeleton-associated protein 4* | 1.98 | 1.58E-02 | 0.09 | NP_006816 | CKAP4 |
| Rhodopsin kinase* | 1.97 | 1.74E-03 | 0.05 | NP_002920 | GRK1 |
| WD repeat-containing protein 1 | 1.96 | 1.58E-02 | 0.11 | NP_059830 | WDR1 |
| Heat shock 70 kDa protein 6 | 1.96 | 1.09E-02 | 0.048 | NP_002146 | HSPA6 |
| Very long-chain specific acyl-CoA dehydrogenase, mitochondrial | 1.94 | 1.27E-02 | 0.11 | NP_001029031 | ACADVL |
| 60S acidic ribosomal protein P2* | 1.93 | 4.24E-02 | 0.26 | NP_000995 | RPLP2 |
| Dihydropyrimidinase-related protein 2* | 1.91 | 1.76E-02 | 0.11 | NP_001184222 | DPYSL2 |
| Eukaryotic translation initiation factor 3* | 1.87 | 2.04E-02 | 0.11 | NP_057175 | EIF3L |
| Serine/threonine-protein phosphatase 2A 65 kDa* | 1.87 | 1.07E-02 | 0.49 | NP_055040 | PPP2R1A |
| Stress-induced-phosphoprotein 1 | 1.86 | 1.90E-02 | 0.09 | NP_006810 | STIP1 |
| Arginine-tRNA ligase, cytoplasmic | 1.82 | 2.45E-02 | 0.13 | NP_002878 | RARS |
| T-complex protein 1 | 1.80 | 4.39E-02 | 0.34 | NP_036205 | CCT5 |
| Elongation factor 2* | 1.78 | 4.27E-02 | 0.98 | NP_001952 | EEF2 |
| Coatomer* | 1.78 | 2.60E-02 | 0.03 | NP_001646 | ARCN1 |
| T-complex protein 1* | 1.75 | 4.09E-02 | 0.13 | NP_005989 | CCT3 |
| Heterogeneous nuclear ribonucleoprotein K* | 1.71 | 4.37E-03 | 0.09 | NP_112553 | HNRNPK |
| Zinc finger protein 292* | 1.71 | 1.06E-02 | 0.13 | NP_055836 | ZNF292 |
| Death-inducer obliterator 1 | 1.70 | 4.62E-02 | 0.28 | NP_149072 | DIDO1 |
| Mitotic spindle assembly checkpoint protein MAD1* | 1.69 | 1.42E-03 | 0.04 | NP_003541 | MAD1L1 |
| Afadin* | 1.68 | 6.24E-04 | 0.03 | NP_001035089 | MLLT4 |
| Connector enhancer of kinase suppressor of ras 2 | 1.62 | 2.53E-02 | 0.22 | NP_055742 | CNKSR2 |
| Protein Shroom 4* | 1.62 | 2.28E-02 | 0.21 | NP_065768 | SHROOM4 |
| 60S ribosomal protein L9* | 1.61 | 3.80E-02 | 0.26 | NP_000652 | RPL9 |
| Fez family zinc finger protein 1* | 1.61 | 4.65E-02 | 0.28 | NP_001019784 | FEZF1 |
| Poly [ADP-ribose] polymerase 4 | 1.58 | 1.11E-02 | 0.13 | NP_006428 | PARP4 |
| Glycogen phosphorylase, muscle form | 1.57 | 3.92E-04 | 0.02 | NP_001158188 | PYGM |
| Peroxisome proliferator-activated receptor gamma coactivator-related protein 1* | 1.54 | 8.25E-03 | 0.12 | NP_055877 | PPRC1 |
| Nuclear pore complex protein Nup98-Nup96* | 1.54 | 5.19E-04 | 0.02 | NP_624358 | NUP98 |
| TBC1 domain family member 2A* | 1.53 | 6.99E-04 | 0.03 | NP_060891 | TBC1D2 |
| Transcriptional regulator ATRX* | 1.52 | 6.45E-03 | 0.11 | NP_000480 | ATRX |
| Tryptophan-tRNA ligase, cytoplasmic | 1.52 | 1.94E-02 | 0.19 | NP_004175 | WARS |
| Fermitin family homolog 2 | 1.52 | 1.49E-02 | 0.16 | NP_006823 | FERMT2 |
| Thioredoxin | 1.52 | 2.17E-02 | 0.20 | NP_003320 | TXN |
| Lipid phosphate phosphatase-related protein type 4* | 1.51 | 6.02E-03 | 0.11 | NP_055654 | LPPR4 |

**Table S2:** Pathway analysis of proteins listed in Table S1. (a) Top networks, (b) molecular and cellular functions, The functional protein association network analysis was carried out with STRING version 10.0 and Ingenuity Pathway Analysis software version 01-07 and gave consistent functional classifications of the proteins of interest as presented in (a-e).

**(a) Top Networks (IPA)**

| **ID Associated Network Functions** | **Score** |
| --- | --- |
| Cellular Assembly and Organization, Post-Translational Modification, Protein Folding | 62 |
| Cancer, Gastrointestinal Disease, Organismal Injury and Abnormalities | 28 |
| Cancer, Gastrointestinal Disease, Hepatic System Disease | 28 |
| Developmental Disorder, Hereditary Disorder, Metabolic Disease | 25 |
| DNA Replication, Recombination, and Repair, Energy Production, Nucleic Acid Metabolism | 8 |

**(b) Molecular and cellular functions (IPA)**

| **Name** | **p-value** | **#Molecules** |
| --- | --- | --- |
| Cellular Assembly and Organization | 4.86E-02 - 4.13E-05 | 31 |
| Cellular Function and Maintenance | 4.84E-02 - 4.13E-05 | 20 |
| Cell Morphology | 4.86E-02 - 7.43E-05 | 17 |
| Post-Translational Modification | 4.40E-02 - 1.71E-05 | 8 |
| Protein Folding | 4.69E-05 - 1.71E-05 | 5 |

References

1. Zhao L, Ashek A, Wang L, Fang W, Dabral S, Dubois O, Cupitt J, Pullamsetti SS, Cotroneo E, Jones H, Tomasi G, Nguyen QD, Aboagye EO, El-Bahrawy MA, Barnes G, Howard LS, Gibbs JS, Gsell W, He JG, Wilkins MR. Heterogeneity in lung (18)FDG uptake in pulmonary arterial hypertension: potential of dynamic (18)FDG positron emission tomography with kinetic analysis as a bridging biomarker for pulmonary vascular remodeling targeted treatments. *Circulation*; **2013**: 128(11): 1214-1224.

2. Xue X, Lai KT, Huang JF, Gu Y, Karlsson L, Fourie A. Anti-inflammatory activity in vitro and in vivo of the protein farnesyltransferase inhibitor tipifarnib. *J Pharmacol Exp Ther* 2006;**317**:53-60.

3. Du W, Lebowitz PF, Prendergast GC. Cell growth inhibition by farnesyltransferase inhibitors is mediated by gain of geranylgeranylated RhoB. *Mol Cell Biol* 1999;**19**:1831-1840.

4. Abdul-Salam VB, Wharton J, Cupitt J, Berryman M, Edwards RJ, Wilkins MR. Proteomic analysis of lung tissues from patients with pulmonary arterial hypertension. *Circulation* 2010;**122**:2058-2067.
